# Effect of 9p21.3 (lncRNA and CDKN2A/2B) variant on lipid profile

**DOI:** 10.3389/fcvm.2022.946289

**Published:** 2022-09-07

**Authors:** Baozhu Wei, Yang Liu, Hang Li, Yuanyuan Peng, Zhi Luo

**Affiliations:** ^1^Department of Cardiology, Zhongnan Hospital of Wuhan University, Wuhan University, Wuhan, China; ^2^Institute of Myocardial Injury and Repair, Wuhan University, Wuhan, China; ^3^Department of Endocrinology, China Resources and WISCO General Hospital, Wuhan, China; ^4^Department of Gerontology, Zhongnan Hospital of Wuhan University, Wuhan University, Wuhan, China

**Keywords:** lncRNA, CDKN2A/2B, variant, dyslipidemia, coronary artery disease

## Abstract

**Background:**

Several 9p21.3 variants, such as rs1333049, rs4977574, rs10757274, rs10757278, and rs10811661, identified from recent genome-wide association studies (GWASs) are reported to be associated with coronary artery disease (CAD) susceptibility but independent of dyslipidemia. This study investigated whether these 9p21.3 variants influenced lipid profiles.

**Methods and results:**

By searching the PubMed and Cochrane databases, 101,099 individuals were included in the analysis. The consistent finding for the rs1333049 C allele on lipid profiles increased the triglyceride (TG) levels. Moreover, the rs4977574 G allele and the rs10757274 G allele, respectively, increased low-density lipoprotein cholesterol (LDL-C) and high-density lipoprotein cholesterol (HDL-C) levels. However, the rs10811661 C allele largely reduced LDL-C levels. Subgroup analyses indicated that the effects of the rs1333049 C allele, rs4977574 G allele, and rs10757274 G allele on lipid profiles were stronger in Whites compared with Asians. In contrast, the effect of the rs10811661 C allele on lipid profiles was stronger in Asians compared with Whites.

**Conclusion:**

The rs1333049 C allele, rs4977574 G allele, and rs10757274 G allele of lncRNA, and the rs10811661 G allele of CDKN2A/2B had a significant influence on lipid levels, which may help the understanding of the underlying mechanisms between 9p21.3 variants and CAD.

## Introduction

The variant (mutation) of the 9p21.3 allele is prevalent in the general population at a rate of 50% (1–4). One copy of the 9p21.3 allele increases the risk of coronary artery disease (CAD) by 25%, whereas two copies augment the risk by 50% (1–4).

The 9p21.3 allele contains a long non-coding RNA (also known as lncRNA, ANRIL, or CDKN2BAS) and two protein-encoding genes (CDKN2A and CDKN2B). rs1333049, a genetic variant in lncRNA, is formed by a nucleotide substitution from guanine (G) to cytosine (C). In addition, rs4977574, rs10757274, and rs10757278, genetic variants in lncRNA, are formed by a nucleotide substitution from adenine (A) to guanine (G). Moreover, rs10811661, a genetic variant in CDKN2A/2B, is formed by a nucleotide substitution from thymine (T) to cytosine (C).

A series of basic studies tested the effects of gene expression levels of lncRNA and CDKN2A/2B on lipid profiles, but the results were inconsistent. For instance, the knockdown of lncRNA inhibits lipid uptake and accumulation and promotes macrophage reverse cholesterol transport (mRCT) (5), while the overexpression of lncRNA significantly increases intracellular lipid accumulation in macrophage-derived foam cells (5). In contrast, the knockdown of CDKN2B induced C/EBPα expression and lipid accumulation in 3T3-L1 cells (6). However, knockout of the 9p21 risk locus (chr4Δ70kb/Δ70kb, ApoE–/– on 129 background) (7) or CDKN2A (8) did not affect lipid levels in deficient mice. Similarly, a large body of clinical trials have detected the effects of these five variants on lipid profiles, but the results are controversial. For instance, the C allele of rs1333049 increased triglyceride (TG) (9), total cholesterol (TC) (10), and low-density lipoprotein cholesterol (LDL-C) (9, 10) and lowered high-density lipoprotein cholesterol (HDL-C) levels (9). In contrast, the G allele of rs4977574 increased TC (11) and lowered the HDL-C levels (11), the G allele of rs10757274 increased the HDL-C levels (12), the G allele of rs10757278 increased the TC (13) and LDL-C levels (13), and the C allele of rs10811661 decreased the TG (14–16), TC (14, 15), and LDL-C (15) levels and increased the HDL-C levels (15, 16). Notably, an observational study conducted by Ahmed et al. (17) indicated that the C allele of rs1333049 remodeled lipid metabolism, therefore influencing myocardial infarction risk. Moreover, a set of elegant lipidomics studies (NPHSII, Northwick Park Heart Study II) conducted by Meckelmann et al. (18) indicated that the G allele of rs10757274 remodeled CAD risk by modulating lipid metabolism. However, the results obtained from other clinical trials did not favor these findings (19–23). Hence, a meta-analysis is needed to clarify whether these variants affected lipid profiles to resolve these discrepancies.

It has been well documented that rs1333049 C allele (1), rs4977574 G allele (24), rs10757274 G allele (2), rs10757278 G allele (3), and rs10811661 T allele (25) increased the risk of CAD by 30–40% in Whites (1–3, 25) and East Asians (24, 25). Since dyslipidemia is one of the most important risk factors for CAD and accounts for at least 50% of the population-attributable risk (26), it is tempting to speculate that the increased CAD risk caused by these variants may stem from a remodeled lipid profile. Surprisingly, the increase in CAD risk attributed to these risk alleles was reported to be independent of dyslipidemia in four GWAS studies (1–4). Since dyslipidemia is closely related to the pathogenesis of CAD (26), these five variants indeed have the potential to affect lipid levels (9–18). It is reasonable to speculate that these five variants may affect lipid profiles after enhancing statistical power. Therefore, we conducted this meta-analysis in a large sample size (101,099 individuals) to identify whether these five variants remodeled lipid metabolism and to increase our understanding of the underlying mechanisms between 9p21.3 variants and CAD.

Due to diverse constraints, most independent or single clinical trials are imperfect or flawed. For instance, the sample size is too small, the gender is not balanced, the coverage of the age group is too narrow, or it is impossible to obtain richer and more accurate experimental results due to the limitations of the examining technology or experimental condition (27–29). Since these constraints are inevitable and usually result in severe deviations or heterogeneity, a common truth may be masked by these independent clinical trials (27–29). Therefore, we attempted to utilize the light of evidence-based medicine by a meta-analysis to examine the differences and to identify the sources of heterogeneity across these independent studies to reveal a common truth (whether variants of rs1333049, rs4977574, rs10757274, rs10757278, and rs10811661 in 9p21.3 are statistically impacted or with a trend to influence lipid levels). Moreover, the specific reason to select these five variants rather than other variants loci in 9p21.3, such as LINC-PINT (30), LINC00599 (30), or rs1537373 (31), is due to only these five variants with sufficient data to execute meta-analysis.

In the past few decades, circulating TG levels have been widely reported to be associated with the occurrence, progress, and prognosis of CAD. For instance, a meta-analysis of the Asia-Pacific region indicated that TG was an independent predictor of CAD (32). This is consistent with the findings of a Chinese multi-provincial cohort study (33). Moreover, another meta-analysis of 29 prospective studies (34) indicated that TG was moderately or highly associated with CAD risk. Intriguingly, it was verified by the Copenhagen City Heart Study (35), whereby increased TG levels were associated with an increased risk of myocardial infarction, ischemic heart disease, and death. Notably, the Bezafibrate Infarction Prevention trial (36) further revealed that increased TG levels were independently associated with increased mortality in patients with CAD, indicating that circulating TG was a critical risk factor for CAD and hypertriglyceridemia should not be ignored in CAD intervention.

Here, we systematically analyzed the effects of the rs1333049 C allele, rs4977574 G allele, rs10757274 G allele, rs10757278 G allele, and rs10811661 C allele in 9p21.3 on lipid profiles in 101,099 individuals by a meta-analysis.

## Materials and methods

The present meta-analysis follows the Preferred Reporting Items for Systematic Reviews and Meta-analyses (PRISMA) (37).

### Literature search

A comprehensive search of the literature was executed from January 5, 2021 to February 15, 2022, by using the PubMed and Cochrane databases. The following keywords were used in the search: (“long non-coding RNA,” “lncRNA,” “ANRIL,” “CDKN2BAS,” “CDKN2A/2B,” “9p21.3,” “rs1333049,” “rs4977574,” “rs10757274,” “rs10757278,” or “rs10811661”), (“mutation,” “variation,” “mutant,” “variant,” or “polymorphism”), and (“lipids,” “lipid metabolism,” “lipoprotein,” “cholesterol,” “circulating lipids,” “blood lipids,” “plasma lipids,” “serum lipids,” or “lipid profile”).

### Inclusion and exclusion criteria

The inclusion criteria were as follows: (1) Articles that detected the effect of the rs1333049 C allele, rs4977574 G allele, rs10757274 G allele, rs10757278 G allele, or rs10811661 C allele on lipid profiles; (2) articles that provided at least one of the four parameters in lipid profiles [TG, total cholesterol (TC), LDL-C, and HDL-C]; (3) articles that provided genotype frequencies of variants of rs1333049, rs4977574, rs10757274, rs10757278, and rs1081166; (4) articles that offered mean lipid levels with standard deviation (SD) or standard errors (SE) by the genotypes; (5) the interventional studies that provided pre-intervention data; and (6) the language of eligible studies restricted to English and Chinese. The exclusion criteria were as follows: (1) articles not related to rs1333049, rs4977574, rs10757274, rs10757278, and rs10811661; (2) articles in which human subjects used lipid-lowering drugs; (3) articles that did not present genotype counts; (4) studies that provided invalid data; (5) pedigree articles; (6) overlapping articles; and (7) abstract, review, case report, meta-analysis, and animal articles.

### Subgroup analysis

Subgroup analysis was conducted on ethnicity/race and disease status. Ethnicity/race includes White, Asian, and other ethnicities. Disease status includes CAD, type 2 diabetes mellitus (T2DM), and healthy subjects. In some studies, the subjects were divided into more than one subpopulation (e.g., the subjects originated from a different gender or a different race). Each subpopulation was regarded as an independent comparison in this study.

### Other items

Since data extraction and analysis, heterogeneity processing, sensitivity analysis, the risk bias test, and the publication bias test were adopted from previous methods, to avoid redundant descriptions, previous publication by Liu et al. (38) provides more details. Moreover, a study conducted by Phani et al. (23) only offered raw lipid data by the genotypes of rs10811661 in their Supplementary material. Therefore, we downloaded and analyzed those raw data by performing a one-way ANOVA (the Kruskal–Wallis test) in SPSS software (version 23.0, Inc., Chicago, IL, United States).

## Results

### Study selection

**FIGURE 1:**
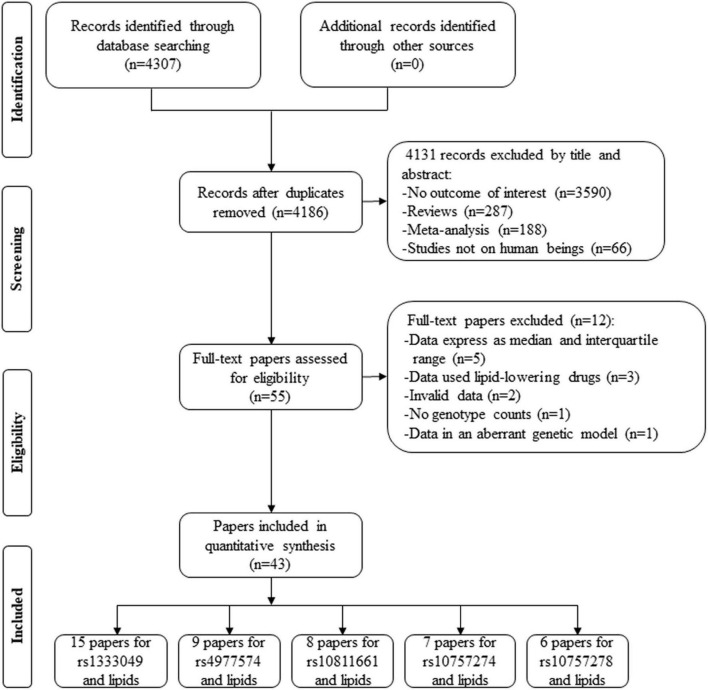
A flow diagram of the study’s selection process.

By searching the PubMed and Cochrane databases, 4,307 articles were identified. After the screening, 4,131 articles were excluded by their title and abstract. Next, 55 articles were further estimated by their contents, of which 5 articles provided lipid data by the genotypes of rs1333049 (39, 40), rs4977574 (41), rs10757274 (42), and rs10811661 (43) but expressed as a median and interquartile range (IQR), 3 articles provided lipid data by the genotypes of rs1333049 but human subjects used lipid-lowering drugs (44–46), 2 articles provided the percentage change of lipid data by the genotypes of rs1333049 (47, 48), 1 article did not present genotype counts of rs1333049 (17), and 1 article (49) provided lipid levels by the genotypes of rs1333049 but in an aberrant genetic model [(CG + GG) vs. CC]. Therefore, 12 articles were further excluded. Finally, 43 articles involving a total of 101,099 individuals were included in the present study (Figure 1). Of the 43 articles, 15 articles (22,513 individuals), 9 articles (31,762 individuals), 7 articles (36,636 individuals), 6 articles (5,156 individuals), and 8 articles (6,028 individuals) were identified for the effects of rs1333049 C allele, rs4977574 G allele, rs10757274 G allele, rs10757278 G allele, and rs10811661 C allele on lipid profiles, respectively.

Characteristics of the included studies are presented in Supplementary Table 1. Circulating lipid levels by the genotypes rs1333049, rs4977574, rs10757274, rs10757278, and rs10811661 are presented in Supplementary Tables 2–6, respectively.

### Effect of rs1333049 C allele on lipid profile

**FIGURE 2:**
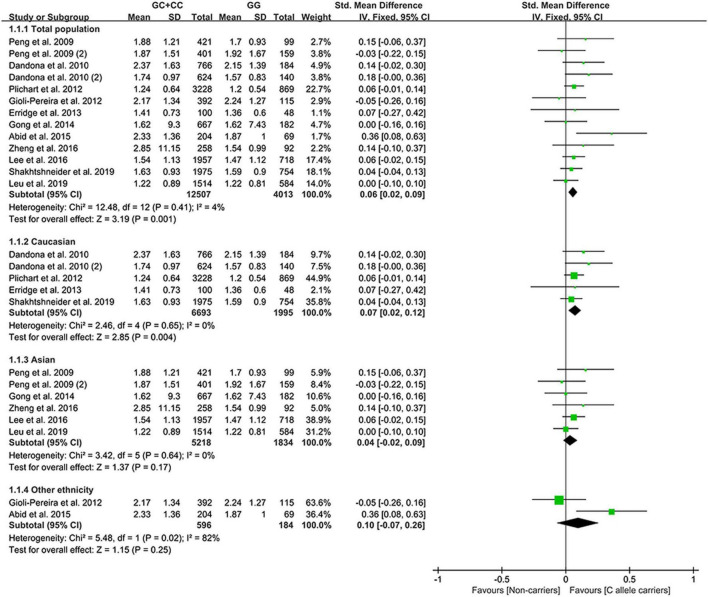
A forest plot of lncRNA rs1333049 variant with circulating TG levels.

All the results stated below were statistically analyzed from the included studies that eliminated heterogeneity (please see “recalculated results that eliminated heterogeneity” in Supplementary Tables 7–11 for more details). The consistent finding for the effects of the rs1333049 C allele on lipid metabolism (Supplementary Table 7 and Supplementary Figure 1) was a slight increase in TG levels (Figure 2). The subgroup analysis indicated that the effect of the rs1333049 C allele on TG levels was observed in Whites (Supplementary Table 7), indicating that Whites with the rs1333049 C allele had an increased risk of CAD. Intriguingly, this speculation was supported by the present analysis results, whereby the C allele of rs1333049 significantly increased the TG levels in patients with CAD (Supplementary Table 7). Meanwhile, the C allele of rs1333049 showed a statistical influence on the TC levels in patients with CAD (Supplementary Table 7).

### Effect of rs4977574 G allele on lipid profile

**FIGURE 3:**
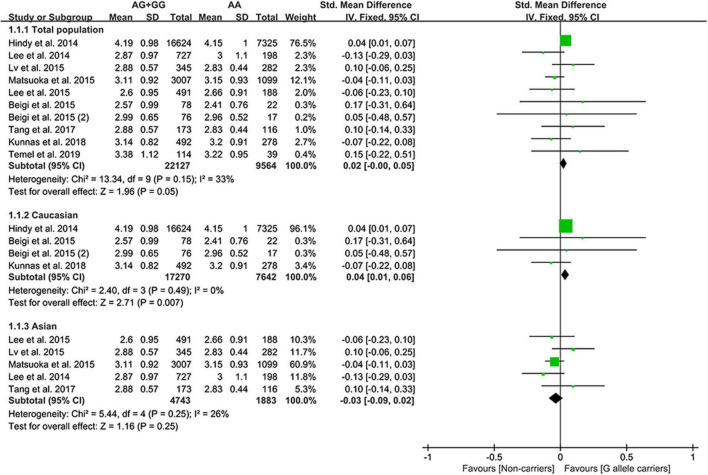
A forest plot of lncRNA rs4977574 variant with circulating LDL-C levels.

The G allele of rs4977574 slightly increased the LDL-C (Figure 3) levels and lowered the TG levels (Supplementary Figure 2). Subgroup analyses indicated that the effects of the rs4977574 G allele on the LDL-C, HDL-C, and TG levels were noted in Whites and healthy subjects (Supplementary Table 8). This indicates that Caucasians and healthy subjects with the G allele of rs4977574 were at a high risk of CAD due to the increased LDL-C levels.

### Effect of rs10757274 G allele on lipid profile

**FIGURE 4:**
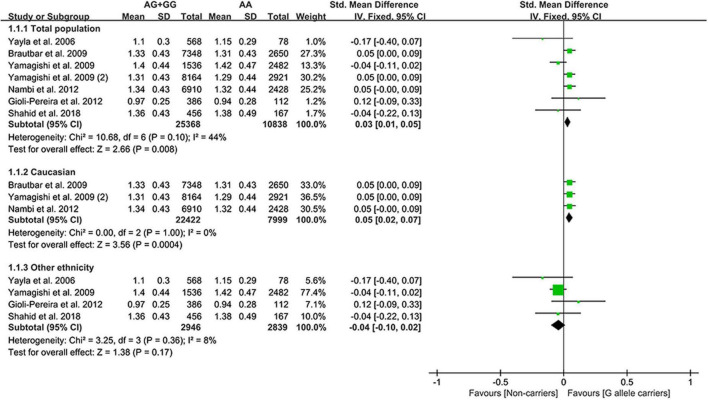
A forest plot of lncRNA rs10757274 variant with circulating HDL-C levels.

The G allele of rs10757274 slightly increased HDL-C (Figure 4) and lowered TG levels (Supplementary Figure 3). Subgroup analyses indicated that the effects of the rs10757274 G allele on the HDL-C and TG levels were noted in Whites and healthy subjects (Supplementary Table 9). This indicates that Whites and healthy subjects with the G allele of rs10757274 may have a reduced CAD risk.

### Effect of rs10757278 G allele on lipid profile

The present study indicated that the G allele of rs10757278 did not statistically influence lipid levels (Supplementary Figure 4 and Supplementary Table 10).

### Effect of rs10811661 C allele on lipid profile

**FIGURE 5:**
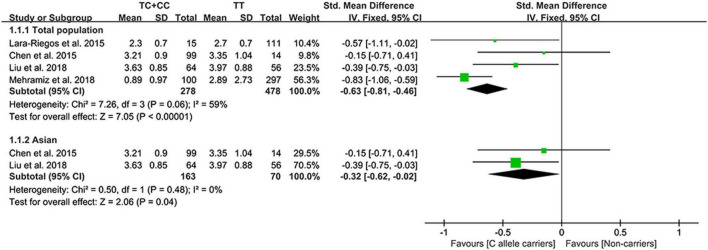
A forest plot of lncRNA rs10811661 variant with circulating LDL-C levels.

The C allele of rs10811661 significantly reduced the LDL-C (Figure 5) and TC levels (Supplementary Figure 5). Subgroup analyses indicated that the significant effects of the rs10811661 C allele on the LDL-C and TC levels were observed in Asians and in patients with T2DM (Supplementary Table 11). This indicates that Asians and patients with T2DM with the C allele of rs10811661 were at low risk of CAD. Moreover, the C allele of rs10811661 significantly reduced the TG levels in Whites (Supplementary Table 11), indicating that Whites with the rs10811661 C allele may have a reduced risk of CAD.

### Evaluation of heterogeneity

Significant heterogeneity was detected in the analysis of the effects of the rs1333049 C allele, the rs4977574 G allele, and the rs10811661 G allele on lipid profiles (Supplementary Tables 7–10). The SMD and 95% CIs of TC and HDL-C modulated by the rs1333049 C allele did not change substantially after eliminating heterogeneity (see Supplementary Table 7 for more details). However, the standardized mean difference (SMD) and 95% *CI* of TG and LDL-C affected by the rs4977574 G allele and the rs10811661 C allele changed significantly after eliminating heterogeneity (as shown in Supplementary Tables 8, 11 for more details).

### Sensitivity analysis

Sensitivity analysis showed that one comparison [Shakhtshneider et al. (19)] may affect the effect of the rs1333049 C allele on the LDL-C levels (Supplementary Figure 6), one comparison [Hindy et al. (50)] may affect the effects of the rs4977574 G allele on the LDL-C and HDL-C levels (Supplementary Figure 7), and one comparison [Mehramiz et al. (16)] may affect the effect of the rs10811661 C allele on the LDL-C levels (Supplementary Figure 8). However, the effects of the rs1333049 C allele, rs4977574 G allele, and rs10811661 C allele on lipid profiles did not change substantially after omitting these comparisons. This indicates that the synthetic results were robust.

### Risk bias test

**FIGURE 6:**
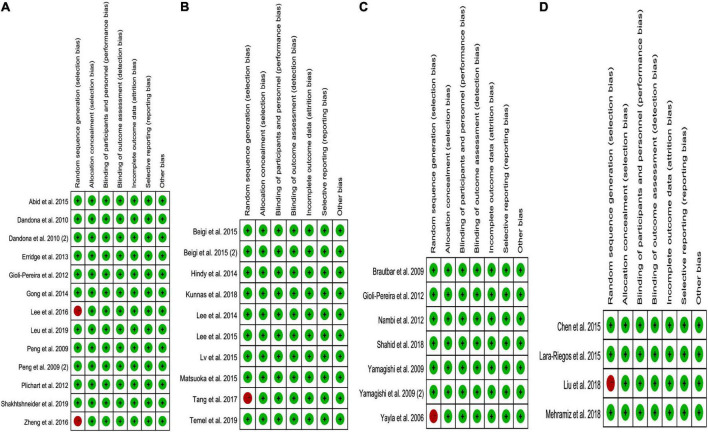
The risk bias plot of lncRNA variant with circulating lipid levels [**(A)** rs1333049 with TG; **(B)** rs4977574 with LDL-C; **(C)** rs10757274 with HDL-C; and **(D)** rs10811661 with LDL-C].

While analyzing the effects of the rs1333049 C allele, rs4977574 G allele, rs10757274 G allele, and rs10811661 C allele on lipid profiles, some concerns were observed in the randomization process (10–25%). However, the overall results showed a low risk of bias (75–90%) among the included studies (Figure 6). Consequently, the studies included in the meta-analysis were of relatively high quality.

### Publication bias test

In the present study, Begg’s test did not find any publication bias. This was confirmed by Egger’s regression test (as seen in Supplementary Figures 9–13 for more details).

## Discussion

In sharp contrast to previous GWAS studies (1–4), our study indicated that the rs1333049 C allele, rs4977574 G allele, rs10757274 G allele, and rs10811661 C allele were robustly associated with lipid profiles in 101,099 individuals. This may help the understanding of the underlying mechanisms between these 9p21.3 variants and CAD.

The precise mechanisms underlying the effect of these five 9p21.3 variants on lipid profiles have not been clarified yet. However, several putative regulatory pathways could be proposed to interpret its mechanisms. (1) By modulating the expression of CDKN2A/2B. CDKN2A/2B plays a crucial role in regulating reverse cholesterol transport (RCT) (5) and in maintaining lipid metabolism homeostasis (6). However, the expression of CDKN2A/2B was largely determined by the C allele of rs1333049 (51) and rs10811661 (52) and the G allele of rs4977574 (53), rs10757274 (54) and rs10757278 (55, 56). Therefore, these five variants may indirectly influence the lipid levels by impacting CDKN2A/2B expression. (2) By modulating the expression of lipid metabolism-related genes. lncRNA is a known regulator of multiple genes (57), which may remodel lipid metabolism by regulating lipid metabolism-related genes (58). Therefore, some genetic variants in 9p21.3, such as rs1333049 C allele (59, 60), rs4977574 G allele (60), rs10757274 G allele (60), rs10757278 G allele (60, 61), and rs10811661 C allele (52), may affect the lipid levels by influencing ANRIL expression. Intriguingly, this speculation was supported by a lipidomics study (18), in which the G allele of rs10757274 remodeled the lipid metabolism by altering the expression of lysophospholipids (LysoPLs), lysophosphatidic acid (LysoPA), and autotaxin (ATX). Notably, other lipid metabolism-related genes, such as, low density lipoprotein receptor (LDLR) (62), very low density lipoprotein receptor (VLDLR) (63), ATP-binding cassette transporter A1 (ABCA1) (64), apolipoprotein C2 (APOC2) (65), apolipoprotein A-I (APOA1) (66), and HMG-CoA reductase (HMGCR) (67) may also be regulated by these variants.

In the present study, the C allele of rs1333049 showed a statistical influence on TG and TC levels in patients with CAD (Supplementary Table 7), indicating the significant association between the rs1333049 C allele and increased CAD risk (1) was mediated, at least partly, by increased TG and TC levels. However, the G allele of rs4977574 simultaneously increased the LDL-C (harmful) levels and lowered the TG (beneficial) levels (Supplementary Table 8), indicating that the rs4977574 G allele had an ambiguous influence on lipid profiles. When combined with a previous GWAS study (24), whereby the G allele of rs4977574 largely increased the risk of CAD, the increased levels of LDL-C at least partly mediated the correlation between the rs4977574 G allele and increased CAD risk.

The G allele of rs10757274 was identified as a risk allele for CAD in the McPherson et al. (2) study. Since the rs10757274 G allele may interact with cigarette smoking, alcohol consumption, the presence of hypertension, the presence of diabetes, and a family history of CAD to modulate CAD risk (68, 69), and the G allele of rs10757274 slightly increased the HDL-C levels and reduced the TG levels in the present study (Supplementary Table 9). This indicates that the correlation between the rs10757274 G allele and increased CAD risk (2) was more likely mediated by other cardiovascular risk factors, such as cigarette smoking, alcohol consumption, the presence of hypertension, the presence of diabetes, and a family history of CAD (68, 69), but not ameliorated lipid metabolism (Supplementary Table 9). Intriguingly, this speculation was favored by a large-sale GWAS study conducted by Angelakopoulou et al. (70), in which the G allele of rs10757274 significantly increased the risk of CAD but independent of dyslipidemia. More large-scale clinical trials are needed to verify this speculation.

However, we did not observe the effects of the rs10757278 G allele on lipid profiles (Supplementary Table 10). Since the G allele of rs10757278 may regulate the expression of CDKN2A/2B (55, 56) and/or ANRIL (60, 61) and was closely related to the onset of myocardial infarction (3), it was reasonable to speculate that the G allele of rs10757278 very likely impacted lipid metabolism. One plausible explanation that can be proposed to interpret this phenomenon is that the sample size included for rs10757278 lipid association analysis was relatively small (as seen in Supplementary Table 10 for more details), which largely reduced the statistical power; therefore, future large-scale clinical trials are needed to verify or correct our findings.

Strikingly, the C allele of rs10811661 substantially reduced the LDL-C and TC levels in Asians (Supplementary Table 11), indicating that the correlation between the rs10811661 C allele and reduced CAD risk in Asians (25) was mediated, at least partly, by decreased LDL-C and TC levels. According to American College of Cardiology/American Heart Association (ACC/AHA) (71), European Society of Cardiology/European Atherosclerosis Society (ESC/EAS) (72), and the adult treatment panel III (ATP III) cholesterol guidelines (73), LDL-C was considered the major cause of CAD and was treated as the primary target for therapy, while other lipids were used as the secondary or supplementary therapeutic targets. Since rs10811661 C allele largely reduced the LDL-C levels, the C allele of rs10811661 may be a potential marker for dyslipidemia and/or CAD.

According to the JDSC study (74), TG was considered a risk factor for CAD comparable with LDL-C (a 1-mmol/L increase in the baseline TG and LDL-C levels were associated with 63 and 64% higher risk of CAD, respectively). In the present study, the C allele of rs1333049, the G allele of rs4977574, and the G allele of rs10757274 consistently affected TG levels (Supplementary Tables 7–9). This indicates the effects of the rs1333049 C allele, rs4977574 G allele, and rs10757274 G allele on lipid profiles predominantly in the TG levels. Since TG levels were closely related to the pathogenesis of CAD (30–34, 71), these three alleles of the three variants may be a potential marker for CAD.

### Strengths and limitations

To the best of our knowledge, this is the first reliable evidence that demonstrates that the rs1333049 C allele, rs4977574 G allele, rs10757274 G allele, and rs10811661 C allele in 9p21.3 had a statistical influence on lipid profiles. Several strengths of the present study should be noted. For instance, the clinical lipid data of 101,099 individuals were included in the analysis, which increased the reliability of synthetic results due to high statistical power. Moreover, data analyses were performed after eliminating the studies with heterogeneity, which further advanced the preciseness of conclusions drawn in our study. Most importantly, our findings may help the understanding of the underlying mechanisms between variants of rs1333049, rs4977574, rs10757274, and rs10811661 in 9p21.3 and CAD. However, a large number of genes as well as some environmental factors are involved in dyslipidemia. Our study has not investigated the interaction of the 9p21.3 variant with other variant locus or environmental factors on lipid profiles due to the lack of original data from the included studies.

## Conclusion

The rs1333049 C allele, rs4977574 G allele, rs10757274 G allele of lncRNA and the rs10811661 G allele of CDKN2A/2B had a significant influence on lipid levels, which may help the understanding of the underlying mechanisms between 9p21.3 variants and CAD.

## Data availability statement

The original contributions presented in this study are included in the article/Supplementary material, further inquiries can be directed to the corresponding authors.

## Author contributions

ZL, BW, and YL conceived and designed this study as well as drafted the manuscript. HL and YP carried out the searches and collected the data. ZL performed the statistical analyses. All authors reviewed and approved the final manuscript.
