# Supplementary material for: Effect of 9p21.3 (lncRNA and CDKN2A/2B) variant on lipid profile
Source: Front Cardiovasc Med. 2022 Sep 7;9:946289. doi: 10.3389/fcvm.2022.946289 (PMC9489913; doi:10.3389/fcvm.2022.946289)
Supplement: Supplementary file 1 [file Data_Sheet_1.docx]

Supplementary Material

**Supplemental Tables:**

**Table S1.** Characteristics of the included studies.

**Table S2.** Circulating lipid levels by the genotypes of lncRNA rs1333049 variant.

**Table S3.** Circulating lipid levels by the genotypes of lncRNA rs4977574 variant.

**Table S4.** Circulating lipid levels by the genotypes of lncRNA rs10757274 variant.

**Table S5.** Circulating lipid levels by the genotypes of lncRNA rs10757278 variant.

**Table S6.** Circulating lipid levels by the genotypes of CDKN2A/2B rs10811661 variant.

**Table S7.** Meta-analysis of lncRNA rs1333049 variant with circulating lipid levels.

**Table S8.** Meta-analysis of lncRNA rs4977574 variant with circulating lipid levels.

**Table S9.** Meta-analysis of lncRNA rs10757274 variant with circulating lipid levels.

**Table S10.** Meta-analysis of lncRNA rs10757278 variant with circulating lipid levels.

**Table S11.** Meta-analysis of CDKN2A/2B rs10811661 variant with circulating lipid levels.

**Supplemental Figures:**

**Figure S1.** Forest plot of lncRNA rs1333049 variant with circulating TC, LDL-C and HDL-C levels.

**Figure S2.** Forest plot of lncRNA rs4977574 variant with circulating TG, TC and HDL-C levels.

**Figure S3.** Forest plot of lncRNA rs10757274 variant with circulating TG, TC and LDL-C levels.

**Figure S4.** Forest plot of lncRNA rs10757278 variant with circulating lipid levels.

**Figure S5.** Forest plot of CDKN2A/2B rs10811661 variant with circulating TC, TG and HDL-C levels.

**Figure S6.** Sensitivity analysis between lncRNA rs1333049 variant and circulating lipid levels.

**Figure S7.** Sensitivity analysis between lncRNA rs4977574 variant and circulating lipid levels.

**Figure S8.** Sensitivity analysis between CDKN2A/2B rs10811661 variant and circulating lipid levels.

**Figure S9.** Begg’s funnel plot of the effects of lncRNA rs1333049 variant and circulating lipid levels.

**Figure S10.** Begg’s funnel plot of the effects of lncRNA rs4977574 variant and circulating lipid levels.

**Figure S11.** Begg’s funnel plot of the effects of lncRNA rs10757274 variant and circulating lipid levels.

**Figure S12.** Begg’s funnel plot of the effects of lncRNA rs10757278 variant and circulating lipid levels.

**Figure S13.** Begg’s funnel plot of the effects of CDKN2A/2B rs10811661 variant and circulating lipid levels.

**Table S1.** Characteristics of the included studies.

| **First author, reference** | **Year** | **Ethnicity** | **Gender** | **Study population** | **Outcomes** |
| --- | --- | --- | --- | --- | --- |
| Yayla Ç [R1] | 2006 | Other ethnicity | M/F | Patients with CAD and control subjects | TC/LDL-C/HDL-C |
| Ye S [R2] | 2008 | Caucasian | M/F | Healthy subjects | LDL-C/HDL-C |
| Brautbar A [R3] | 2009 | Caucasian | M/F | Healthy subjects | TG/TC/LDL-C/HDL-C |
| Yamagishi K1 [R4] | 2009 | Other ethnicity ns | M/F | Healthy subjects | TC/HDL-C |
| Yamagishi K2 [R4] | 2009 | Caucasian | M/F | Healthy subjects | TC/HDL-C |
| Peng WH1 [R5] | 2009 | Asian | M/F | Patients with CAD | TG/TC/LDL-C/HDL-C |
| Peng WH2 [R5] | 2009 | Asian | M/F | Healthy subjects | TG/TC/LDL-C/HDL-C |
| Wen J1 [R6] | 2010 | Asian | M/F | Patients with T2DM | TG |
| Wen J2 [R6] | 2010 | Asian | M/F | Healthy subjects | TG |
| Patel RS [R7] | 2010 | Caucasian | M/F | Patients with CAD | TC/LDL-C/HDL-C |
| Ye S [R8] | 2010 | Caucasian | M/F | Healthy subjects | LDL-C/HDL-C |
| Dandona S1 [R9] | 2010 | Caucasian | M/F | Patients with CAD | TG/TC/LDL-C/HDL-C |
| Dandona S2 [R9] | 2010 | Caucasian | M/F | Patients with CAD | TG/TC/LDL-C/HDL-C |
| Nambi V [R10] | 2012 | Caucasian | M/F | Healthy subjects | TG/TC/LDL-C/HDL-C |
| Plichart M [R11] | 2012 | Caucasian | M/F | Healthy subjects | TG/TC/HDL-C |
| Gioli-Pereira L [R12] | 2012 | Other ethnicity | M/F | Patients with CAD | TG/TC/LDL-C/HDL-C |
| Erridge C [R13] | 2013 | Caucasian | M | Healthy subjects | TG/LDL-C/HDL-C |
| Gong L [R14] | 2014 | Asian | M/F | Patients with CAD and control subjects | TG/TC/LDL-C/HDL-C |
| Hindy G [R15] | 2014 | Caucasian | M/F | Healthy subjects | TG/LDL-C/HDL-C |
| Lee IT [R16] | 2014 | Asian | M/F | Patients with CAD | TG/TC/LDL-C/HDL-C |
| Lara-Riegos JC [R17] | 2015 | Other ethnicity | M/F | Patients with T2DM | TC/LDL-C/HDL-C |
| Chen Y [R18] | 2015 | Asian | M/F | Patients with T2DM | TG/TC/LDL-C/HDL-C |
| Bi J [R19] | 2015 | Asian | M/F | Patients with ischemic stroke | TG/TC/LDL-C |
| Abid K [R20] | 2015 | Other ethnicity | M/F | Patients with CAD and control subjects | TG/TC/LDL-C/HDL-C |
| Lv J P [R21] | 2015 | Asian | M/F | Patients with CAD and control subjects | LDL-C |
| Matsuoka R [R22] | 2015 | Asian | M/F | Patients with CAD and control subjects | TG/LDL-C/HDL-C |
| Lee IT [R23] | 2015 | Asian | M/F | Patients with angina pectoris | TG/TC/LDL-C/HDL-C |
| Beigi SS1 [R24] | 2015 | Caucasian | M/F | Patients with CAD | TG/TC/LDL-C/HDL-C |
| Beigi SS2 [R24] | 2015 | Caucasian | M/F | Control subjects | TG/TC/LDL-C/HDL-C |
| Phani NM [R25] | 2016 | Other ethnicity | M/F | Patients with T2DM and control subjects | TG/TC/LDL-C/HDL-C |
| Zhao Q1 [R26] | 2016 | Asian | M | Patients with CAD and control subjects | TG/TC/LDL-C/HDL-C |
| Zhao Q2 [R26] | 2016 | Asian | F | Patients with CAD and control subjects | TG/TC/LDL-C/HDL-C |
| Zheng C [R27] | 2016 | Asian | M/F | Patients with gout and control subjects | TG/TC/LDL-C/HDL-C |
| Lee CJ [R28] | 2016 | Asian | M/F | Patients with high risk of CAD | TG/TC/HDL-C |
| Shendy HA1 [R29] | 2017 | Caucasian | M/F | Patients with CAD | TC |
| Shendy HA2 [R29] | 2017 | Caucasian | M/F | Patients with CAD | TC |
| Shendy HA3 [R29] | 2017 | Caucasian | M/F | Healthy subjects | TC |
| Tang O [R30] | 2017 | Asian | M/F | Patients with CAD | LDL-C |
| Liu J [R31] | 2018 | Asian | M/F | Patients with gestational diabetes mellitus | TG/TC/LDL-C/HDL-C |
| Mehramiz M1 [R32] | 2018 | Caucasian | M/F | Healthy subjects | TG/TC/LDL-C/HDL-C |
| Mehramiz M2 [R32] | 2018 | Caucasian | M/F | Patients with overweight | TG/TC/LDL-C/HDL-C |
| Mehramiz M3 [R32] | 2018 | Caucasian | M/F | Patients with obesity | TG/TC/LDL-C/HDL-C |
| Plengvidhya N [R33] | 2018 | Other ethnicity | M/F | Patients with T2DM | TC |
| Mahdavi S [R34] | 2018 | Other ethnicity | M/F | Healthy subjects | TG/LDL-C/HDL-C |
| Shahid SU [R35] | 2018 | Other ethnicity | M/F | Patients with CAD and control subjects | TG/TC/LDL-C/HDL-C |
| Kunnas T [R36] | 2018 | Caucasian | M/F | Patients with hypertension and control subjects | TG/TC/LDL-C/HDL-C |
| Temel ŞG1 [R37] | 2019 | Other ethnicity | M/F | Patients with CAD | TG/TC/LDL-C/HDL-C |
| Temel ŞG2 [R37] | 2019 | Other ethnicity | M/F | Control subjects | TG/TC/LDL-C/HDL-C |
| Shakhtshneider E [R38] | 2019 | Caucasian | M/F | Healthy subjects | TG/TC/LDL-C/HDL-C |
| Leu HB [R39] | 2019 | Asian | M/F | Healthy subjects | TG/TC/LDL-C/HDL-C |
| Shakhtshneider E [R40] | 2019 | Caucasian | M/F | Healthy subjects | TG/TC/LDL-C/HDL-C |
| Jacobson P1 [R41] | 2020 | Caucasian | M/F | Patients with obesity | TC/HDL-C |
| Jacobson P2 [R41] | 2020 | Caucasian | M/F | Patients with obesity | TC/HDL-C |
| He SQ [R42] | 2021 | Asian | M/F | Patients with T2DM | TG/TC/LDL-C/HDL-C |
| Bogari N [R43] | 2021 | Caucasian | M/F | Patients with CAD | TG/TC/LDL-C/HDL-C |

M: male; F: female; CAD: coronary artery disease; T2DM: type 2 diabetes mellitus; TG: triglycerides; TC: total cholesterol; LDL-C: low-density lipoprotein cholesterol;

HDL-C: high-density lipoprotein cholesterol.

**Table S2.** Circulating lipid levels by the genotypes of lncRNA rs1333049 variant.

| **First author, reference** | **Number** | |  | **TG, mmol/L** | | ***P* value** | **TC, mmol/L** | | ***P* value** | **LDL-C, mmol/L** | | ***P* value** | **HDL-C, mmol/L** | | ***P* value** |
| --- | --- | --- | --- | --- | --- | --- | --- | --- | --- | --- | --- | --- | --- | --- | --- |
|  | **GG** | **GC+CC** |  | **GG** | **GC+CC** |  | **GG** | **GC+CC** |  | **GG** | **GC+CC** |  | **GG** | **GC+CC** |  |
| Ye S [R2] | 100 | 277 |  | - | - | - | - | - | - | 3.65±0.93 | 3.79±1.00 | 0.226 | 1.53±0.44 | 1.52±0.42 | 0.534 |
| Peng WH1 [R5] | 99 | 421 |  | 1.70±0.93 | 1.88±1.21 | - | 4.57±0.94 | 4.61±1.06 | - | 2.67±0.74 | 2.68±0.84 | - | 1.09±0.22 | 1.09±0.25 | - |
| Peng WH2 [R5] | 159 | 401 |  | 1.92±1.67 | 1.87±1.51 | - | 4.63±1.03 | 4.61±0.98 | - | 2.61±0.80 | 2.59±0.82 | - | 1.24±0.43 | 1.32±0.48 | - |
| Ye S [R8] | 137 | 401 |  | - | - | - | - | - | - | 3.47±0.87 | 3.54±0.89 | 0.724 | 1.66±0.36 | 1.66±0.37 | 0.645 |
| Dandona S1 [R9] | 184 | 766 |  | 2.15±1.39 | 2.37±1.63 | 0.281 | 5.80±1.34 | 5.94±1.36 | 0.016 | 3.69±1.18 | 3.74±1.13 | 0.047 | 1.13±0.31 | 1.17±0.57 | 0.915 |
| Dandona S2 [R9] | 140 | 624 |  | 1.57±0.83 | 1.74±0.97 | 0.278 | 5.45±1.22 | 5.58±1.25 | 0.689 | 3.52±1.06 | 3.50±0.97 | 0.895 | 1.21±0.28 | 1.23±0.38 | 0.981 |
| Plichart M [R11] | 869 | 3228 |  | 1.20±0.54 | 1.24±0.64 | 0.640 | 5.92±0.95 | 5.92±1.00 | 0.850 | - | - | - | 1.65±0.41 | 1.63±0.41 | 0.190 |
| Gioli-Pereira L [R12] | 115 | 392 |  | 2.24±1.27 | 2.17±1.34 | 0.870 | 5.67±1.29 | 5.82±1.26 | 0.520 | 3.75±1.18 | 3.83±1.13 | 0.600 | 0.93±0.23 | 0.97±0.28 | 0.140 |
| Erridge C [R13] | 48 | 100 |  | 1.36±0.60 | 1.41±0.73 | 0.072 | - | - | - | 2.20±0.60 | 2.29±0.61 | 0.194 | 1.36±0.28 | 1.30±0.26 | 0.173 |
| Gong L [R14] | 182 | 667 |  | 1.62±7.43 | 1.62±9.30 | - | 4.64±1.19 | 4.59±1.19 | - | 2.53±0.96 | 2.46±0.88 | - | 1.13±0.37 | 1.12±0.33 | - |
| Abid K [R20] | 69 | 204 |  | 1.87±1.00 | 2.33±1.36 | 0.001 | 4.89±1.17 | 4.68±1.10 | 0.081 | 3.53±1.34 | 3.80±1.48 | 0.028 | 1.37±0.65 | 1.19±0.46 | 0.060 |
| Zheng C [R27] | 92 | 258 |  | 1.54±0.99 | 2.85±11.15 | 0.669 | 5.18±1.15 | 7.6±30.45 | 0.433 | 3.19±1.00 | 4.50±14.14 | 0.089 | 1.34±0.71 | 1.23±0.33 | 0.142 |
| Lee CJ [R28] | 718 | 1957 |  | 1.47±1.12 | 1.54±1.13 | 0.130 | 5.07±1.16 | 5.08±1.07 | 0.940 | - | - | - | 1.15±0.29 | 1.13±0.30 | 0.610 |
| Shakhtshneider E [R38] | 754 | 1975 |  | 1.59±0.90 | 1.63±0.93 | 0.386 | 5.58±1.35 | 6.48±1.42 | 0.825 | 3.32±1.21 | 3.23±1.24 | 0.173 | 1.54±0.43 | 1.55±0.47 | 0.937 |
| Leu HB [R39] | 584 | 1514 |  | 1.22±0.81 | 1.22±0.89 | - | 5.09±1.09 | 5.08±1.11 | - | 3.35±0.95 | 3.39±1.22 | - | 1.10±0.52 | 1.11±0.34 | - |
| Shakhtshneider E [R40] | 398 | 1025 |  | - | - | - | 6.44±1.34 | 6.40±1.31 | - | - | - | - | - | - | - |
| Jacobson P1 [R41] | 555 | 1297 |  | - | - | - | 5.90±1.10 | 5.80±1.10 | - | - | - | - | 1.40±0.30 | 1.30±0.30 | - |
| Jacobson P2 [R41] | 562 | 1241 |  | - | - | - | 5.70±1.00 | 5.60±1.10 | - | - | - | - | 1.30±0.30 | 1.40±0.30 | - |

lncRNA: long non-coding RNA; TG: triglycerides; TC: total cholesterol; LDL-C: low-density lipoprotein cholesterol; HDL-C: high-density lipoprotein cholesterol.

**Table S3.** Circulating lipid levels by the genotypes of lncRNA rs4977574 variant.

| **First author, reference** | **Number** | |  | **TG, mmol/L** | | ***P* value** | **TC, mmol/L** | | ***P* value** | **LDL-C, mmol/L** | | ***P* value** | **HDL-C, mmol/L** | | ***P* value** |
| --- | --- | --- | --- | --- | --- | --- | --- | --- | --- | --- | --- | --- | --- | --- | --- |
|  | **AA** | **AG+GG** |  | **AA** | **AG+GG** |  | **AA** | **AG+GG** |  | **AA** | **AG+GG** |  | **AA** | **AG+GG** |  |
| Hindy G [R15] | 7325 | 5326 |  | 1.35±0.73 | 1.33±0.77 | 0.480 | - | - | - | 4.15±1.00 | 4.19± 0.98 | 0.330 | 1.39±0.37 | 1.40±0.37 | 0.850 |
| Lee IT [R16] | 198 | 518 |  | 1.60±1.60 | 1.57±1.16 | 0.237 | 4.80±1.30 | 4.70±1.13 | 0.520 | 3.00±1.10 | 2.87±0.97 | 0.275 | 1.10±0.30 | 1.17±0.30 | 0.198 |
| Lv J [R21] | 282 | 2105 |  | - | - | - | - | - | - | 2.83±0.44 | 2.88±0.57 | - | - | - | - |
| Matsuoka R [R22] | 1099 | 1321 |  | 1.58±1.03 | 1.59±1.16 | 0.411 | - | - | - | 3.15±0.93 | 3.11±0.92 | 0.164 | 1.34±0.42 | 1.32±0.39 | 0.762 |
| Lee IT [R23] | 188 | 191 |  | 1.67±2.21 | 1.41±0.86 | 0.077 | 4.55±1.34 | 4.40±1.10 | 0.229 | 2.66±0.91 | 2.60±0.95 | 0.573 | 1.16±0.31 | 1.16±0.36 | 0.170 |
| Beigi SS1 [R24] | 22 | 78 |  | 1.74±0.77 | 1.57±0.85 | 0.700 | 4.34±1.05 | 4.56±1.18 | 0.600 | 2.41±0.76 | 2.57±0.99 | 0.730 | 1.05±0.24 | 1.09±0.27 | 0.860 |
| Beigi SS2 [R24] | 17 | 268 |  | 2.16±0.71 | 2.03±0.62 | 0.370 | 4.93±0.58 | 5.00±0.78 | 0.890 | 2.96±0.52 | 2.99±0.65 | 0.980 | 1.02±0.13 | 1.09±0.16 | 0.060 |
| Tang O [R30] | 116 | 415 |  | - | - | - | - | - | - | 2.83±0.44 | 2.88±0.57 | 0.083 | - | - | - |
| Kunnas T [R36] | 278 | 147 |  | 1.41±0.93 | 1.35±0.90 | 0.665 | 5.40±0.99 | 5.35±0.89 | 0.688 | 3.20±0.91) | 3.14±0.82 | 0.667 | 1.60±0.45 | 1.61±0.45 | 0.823 |
| Temel ŞG1 [R37] | 24 | 90 |  | 1.02±0.22 | 1.46±0.65 | 0.305 | 5.51±1.06 | 5.53±1.43 | 0.019 | 2.64±0.52 | 3.33±0.91 | 0.109 | 1.45±0.33 | 1.30±0.27 | 0.006 |
| Temel ŞG2 [R37] | 39 | 207 |  | 1.14±0.51 | 1.34±0.47 | 0.236 | 5.06±0.99 | 5.08±1.48 | 1.000 | 3.22±0.95 | 3.38±1.12 | 0.480 | 1.37±0.28 | 1.43±0.36 | 0.746 |

lncRNA: long non-coding RNA; TG: triglycerides; TC: total cholesterol; LDL-C: low-density lipoprotein cholesterol; HDL-C: high-density lipoprotein cholesterol.

**Table S4.** Circulating lipid levels by the genotypes of lncRNA rs10757274 variant.

| **First author, reference** | **Number** | |  | **TG, mmol/L** | | ***P* value** | **TC, mmol/L** | | ***P* value** | **LDL-C, mmol/L** | | ***P* value** | **HDL-C, mmol/L** | | ***P* value** |
| --- | --- | --- | --- | --- | --- | --- | --- | --- | --- | --- | --- | --- | --- | --- | --- |
|  | **AA** | **AG+GG** |  | **AA** | **AG+GG** |  | **AA** | **AG+GG** |  | **AA** | **AG+GG** |  | **AA** | **AG+GG** |  |
| Yayla Ç [R1] | 78 | 568 |  | - | - | - | 4.91±1.26 | 5.07±1.26 | 0.210 | 3.04±0.95 | 3.15±1.04 | 0.470 | 1.15±0.29 | 1.10±0.3 | 0.370 |
| Brautbar A [R3] | 2650 | 7348 |  | 1.54±1.00 | 1.52±1.02 | 0.520 | 5.54±1.01 | 5.54±1.06 | 0.350 | 3.54±0.95 | 3.53±0.98 | 0.560 | 1.31±0.43 | 1.33±0.43 | 0.470 |
| Nambi V [R10] | 2428 | 6910 |  | 1.28±0.91 | 1.24±0.88 | - | 5.53±1.01 | 5.52±1.05 | - | 3.54±0.96 | 3.53±0.97 | - | 1.32±0.44 | 1.34±0.43 | - |
| Gioli-Pereira L [R12] | 112 | 386 |  | 2.23±1.27 | 2.20±1.35 | 0.960 | 5.68±1.25 | 5.81±1.29 | 0.580 | 3.74±1.18 | 3.83±1.15 | 0.440 | 0.94±0.28 | 0.97±0.25 | 0.470 |
| Yamagishi K1 [R24] | 2482 | 1536 |  | - | - | - | 5.56±1.16 | 5.53±1.19 | 0.970 | - | - | - | 1.42±0.47 | 1.40±0.44 | 0.300 |
| Yamagishi K2 [R24] | 2921 | 8164 |  | - | - | - | 5.56±1.01 | 5.55±1.05 | 0.390 | - | - | - | 1.29±0.44 | 1.31±0.43 | 0.710 |
| Zhao Q1 [R26] | 23 | 252 |  | 1.64±0.16 | 1.81±0.15 | 0.867 | 3.68±0.21 | 3.98±0.12 | 0.410 | 1.79±0.13 | 1.87±0.08 | 0.915 | 0.98±0.05 | 1.11±0.02 | 0.055 |
| Zhao Q2 [R26] | 11 | 144 |  | 1.99±0.34 | 1.78±0.02 | 0.276 | 4.82±0.19 | 4.29±0.13 | 0.248 | 2.33±0.17 | 1.60±0.42 | 0.326 | 1.23±0.07 | 1.19±0.03 | 0.838 |
| Shahid SU [R35] | 167 | 456 |  | 2.32±0.78 | 2.29±0.79 | 0.794 | 5.09±1.35 | 5.07±1.36 | 0.880 | 2.58±0.73 | 2.54±0.70 | 0.970 | 1.38±0.49 | 1.36±0.43 | 0.188 |

lncRNA: long non-coding RNA; TG: triglycerides; TC: total cholesterol; LDL-C: low-density lipoprotein cholesterol; HDL-C: high-density lipoprotein cholesterol.

**Table S5.** Circulating lipid levels by the genotypes of lncRNA rs10757278 variant.

| **First author, reference** | **Number** | |  | **TG, mmol/L** | | ***P* value** | **TC, mmol/L** | | ***P* value** | **LDL-C, mmol/L** | | ***P* value** | **HDL-C, mmol/L** | | ***P* value** |
| --- | --- | --- | --- | --- | --- | --- | --- | --- | --- | --- | --- | --- | --- | --- | --- |
|  | **AA** | **AG+GG** |  | **AA** | **AG+GG** |  | **AA** | **AG+GG** |  | **AA** | **AG+GG** |  | **AA** | **AG+GG** |  |
| Patel RS [R7] | 557 | 1777 |  | - | - | - | 4.36±1.01 | 4.35±1.14 | 0.400 | 2.50±0.88 | 2.47±0.92 | 0.120 | 1.06±0.33 | 1.06±0.32 | 0.890 |
| Gioli-Pereira L [R12] | 115 | 381 |  | 2.20±1.27 | 2.19±1.35 | 0.990 | 5.69±1.31 | 5.82±1.28 | 0.560 | 3.77±1.23 | 3.84±1.12 | 0.580 | 0.94±0.28 | 0.97±0.26 | 0.200 |
| Bi J [R19] | 38 | 78 |  | 2.23±2.05 | 1.89±1.34 | 0.326 | 4.46±1.06 | 4.73±1.06 | 0.027 | 2.98±0.77 | 3.23±0.80 | 0.013 | - | - | - |
| Shendy HA1 [R29] | 7 | 43 |  | - | - | - | 3.66±0.86 | 5.82±1.19 | 0.073 | - | - | - | - | - | - |
| Shendy HA2 [R29] | 11 | 39 |  | - | - | - | 5.16±0.85 | 4.84±0.67 | 0.410 | - | - | - | - | - | - |
| Shendy HA3 [R29] | 19 | 31 |  | - | - | - | 3.94±0.68 | 3.89±0.7 | 0.890 | - | - | - | - | - | - |
| Mahdavi S [R34] | 454 | 1169 |  | 0.98±2.98 | 0.98±2.33 | 0.990 | - | - | - | 2.27±0.64 | 2.29±0.57 | 0.630 | 1.55±0.43 | 1.53±0.31 | 0.710 |
| Bogari N [R43] | 95 | 342 |  | 2.52±0.73 | 2.52±1.09 | 0.573 | 6.27±1.93 | 6.27±2.57 | 0.827 | 4.46±1.25 | 4.48±1.70 | 0.190 | 1.01±0.76 | 1.02±1.12 | 0.827 |

lncRNA: long non-coding RNA; TG: triglycerides; TC: total cholesterol; LDL-C: low-density lipoprotein cholesterol; HDL-C: high-density lipoprotein cholesterol.

**Table S6.** Circulating lipid levels by the genotypes of CDKN2A/2B rs10811661 variant.

| **First author, reference** | **Number** | |  | **TG, mmol/L** | | ***P* value** | **TC, mmol/L** | | ***P* value** | **LDL-C, mmol/L** | | ***P* value** | **HDL-C, mmol/L** | | ***P* value** |
| --- | --- | --- | --- | --- | --- | --- | --- | --- | --- | --- | --- | --- | --- | --- | --- |
|  | **TT** | **TC+CC** |  | **TT** | **TC+CC** |  | **TT** | **TC+CC** |  | **TT** | **TC+CC** |  | **TT** | **TC+CC** |  |
| Wen J1 [R6] | 409 | 756 |  | 1.70±1.19 | 1.63±1.17 | 0.860 | - | - | - | - | - | - | - | - | - |
| Wen J2 [R6] | 308 | 829 |  | 1.19±0.88 | 1.24±0.86 | 0.240 | - | - | - | - | - | - | - | - | - |
| Lara-Riegos JC [R17] | 111 | 15 |  | - | - | - | 4.60±1.00 | 4.10±0.80 | 0.701 | 2.70±0.70 | 2.30±0.70 | 0.562 | 1.00±0.20 | 0.90±0.20 | 0.414 |
| Chen Y [R18] | 14 | 99 |  | 2.91±1.62 | 2.27±1.09 | 0.01 | 5.49±1.23 | 5.18±1.25 | 0.01 | 3.35±1.04 | 3.21±0.90 | - | 1.18±0.32 | 1.16±0.34 | - |
| Phani NM [R25] | 595 | 562 |  | 1.40±0.49 | 1.42±0.52 | - | 4.52±0.92 | 4.50±0.87 | - | 2.97±1.24 | 2.96±1.05 | - | 0.99±0.31 | 0.99±0.34 | - |
| Liu J [R31] | 56 | 64 |  | 2.39±0.31 | 2.17±0.31 | <0.05 | 5.41±0.89 | 5.33±0.96 | <0.05 | 3.97±0.88 | 3.63±0.85 | <0.05 | 0.81±0.21 | 0.94±0.41 | <0.05 |
| Mehramiz M1 [R32] | 297 | 100 |  | 1.00±0.61 | 0.89±0.57 | - | 4.68±0.96 | 4.65±0.74 | - | 2.89±2.73 | 0.89±0.97 | - | 1.18±0.26 | 1.27±0.28 | <0.05 |
| Mehramiz M2 [R32] | 179 | 66 |  | 1.31±0.85 | 1.16±0.45 | <0.05 | 4.95±0.83 | 4.85±0.85 | - | 3.05±0.87 | 2.96±0.77 | - | 1.16±0.28 | 1.11±0.29 | <0.05 |
| Mehramiz M3 [R32] | 231 | 91 |  | 1.70±0.87 | 1.41±0.86 | <0.05 | 5.02±0.93 | 5.12±0.96 | <0.05 | 2.61±0.88 | 2.76±0.77 | - | 1.12±0.28 | 1.14±0.25 | - |
| Plengvidhya N [R33] | 228 | 272 |  | - | - | 0.197 | 5.25±1.38 | 5.10±1.37 | 0.748 | - | - | 0.943 | - | - | 0.965 |
| He SQ [R42] | 236 | 510 |  | 2.70±2.73 | 2.63±2.22 | 0.807 | 4.96±1.16 | 4.84±1.05 | 0.258 | 2.71±1.01 | 2.69±1.04 | 0.476 | 1.10±0.27 | 1.08±0.28 | 0.575 |

CDKN2A/2B: cyclin-dependent kinase inhibitor 2A/2B; TG: triglycerides; TC: total cholesterol; LDL-C: low-density lipoprotein cholesterol; HDL-C: high-density lipoprotein cholesterol.

**Table S7.** Meta-analysis of lncRNA rs1333049 variant with circulating lipid levels.

| **Groups or subgroups** | **Comparisons**  **(Subjects)** | ***P*_H_** | **SMD (95% CI)** | ***P*_SMD_** |  | **Groups or subgroups** | **Comparisons**  **(Subjects)** | ***P*_H_** | **SMD (95% CI)** | ***P*_SMD_** |
| --- | --- | --- | --- | --- | --- | --- | --- | --- | --- | --- |
| ***Overall results*** | | | | |  | ***Recalculated results that eliminated heterogeneity*** | | | | |
| **TG** |  |  |  |  |  | **TG** |  |  |  |  |
| All | 13 (16 520) | 0.41 | 0.06 (0.02-0.09) | <0.001 |  | All | 13 (16 520) | 0.41 | 0.06 (0.02-0.09) | <0.001 |
| Studies in HWE | 11 (15 608) | 0.38 | 0.05 (0.02-0.09) | <0.01 |  | Studies in HWE | 11 (15 608) | 0.38 | 0.05 (0.02-0.09) | <0.01 |
| Caucasian | 5 (8 688) | 0.65 | 0.07 (0.02-0.12) | <0.01 |  | Caucasian | 5 (8 688) | 0.65 | 0.07 (0.02-0.12) | <0.01 |
| Asian | 6 (7 052) | 0.64 | 0.04 (-0.02-0.09) | 0.17 |  | Asian | 6 (7 052) | 0.64 | 0.04 (-0.02-0.09) | 0.17 |
| Other ethnicity | 2 (780) | 0.02 | 0.10 (-0.07-0.26) | 0.25 |  | Other ethnicity | 2 (780) | 0.02 | 0.10 (-0.07-0.26) | 0.25 |
| CAD | 4 (2 741) | 0.36 | 0.11 (0.02-0.21) | 0.02 |  | CAD | 4 (2 741) | 0.36 | 0.11 (0.02-0.21) | 0.02 |
| Healthy subjects | 5 (9 632) | 0.79 | 0.04 (-0.01-0.08) | 0.12 |  | Healthy subjects | 5 (9 632) | 0.79 | 0.04 (-0.01-0.08) | 0.12 |
| **TC** |  |  |  |  |  | **TC** |  |  |  |  |
| All | 15 (21 450) | <0.001 | 0.05 (-0.08-0.17) | 0.48 |  | All | 13 (18 371) | 0.42 | -0.02 (-0.05-0.02) | 0.34 |
| Studies in HWE | 14 (20 686) | <0.001 | 0.04 (-0.09-0.17) | 0.54 |  | Studies in HWE | 13 (17 957) | 0.49 | -0.02 (-0.05-0.02) | 0.29 |
| Caucasian | 7 (13 618) | <0.001 | 0.09 (-0.14-0.32) | 0.43 |  | Caucasian | 6 (10 889) | 0.14 | -0.03 (-0.07-0.02) | 0.24 |
| Asian | 6 (7 052) | 0.96 | 0.00 (-0.05-0.05) | 0.96 |  | Asian | 6 (7 052) | 0.96 | 0.00 (-0.05-0.05) | 0.96 |
| Other ethnicity | 2 (780) | 0.08 | -0.02 (-0.32-0.28) | 0.89 |  | Other ethnicity | 2 (780) | 0.08 | 0.01 (-0.16-0.17) | 0.94 |
| CAD | 4 (2 741) | 0.96 | 0.10 (0.00-0.19) | 0.05 |  | CAD | 4 (2 741) | 0.96 | 0.10 (0.00-0.19) | 0.05 |
| Healthy subjects | 5 (10 907) | <0.001 | 0.12 (-0.17-0.41) | 0.42 |  | Healthy subjects | 4 (8 178) | 0.98 | -0.01 (-0.06-0.04) | 0.70 |
| **LDL-C** |  |  |  |  |  | **LDL-C** |  |  |  |  |
| All | 13 (10 663) | 0.58 | 0.01 (-0.04-0.05) | 0.78 |  | All | 13 (10 663) | 0.58 | 0.01 (-0.04-0.05) | 0.78 |
| Studies in HWE | 11 (7 276) | 0.46 | 0.01 (-0.04-0.05) | 0.82 |  | Studies in HWE | 11 (7 276) | 0.46 | 0.01 (-0.04-0.05) | 0.82 |
| Caucasian | 6 (4 143) | 0.31 | -0.01 (-0.07-0.05) | 0.70 |  | Caucasian | 6 (4 143) | 0.31 | -0.01 (-0.07-0.05) | 0.70 |
| Asian | 5 (3 261) | 0.71 | 0.01 (-0.06-0.08) | 0.76 |  | Asian | 5 (3 261) | 0.71 | 0.01 (-0.06-0.08) | 0.76 |
| Other ethnicity | 2 (596) | 0.51 | 0.11 (-0.05-0.28) | 0.18 |  | Other ethnicity | 2 (596) | 0.51 | 0.11 (-0.05-0.28) | 0.18 |
| CAD | 4 (2 203) | 0.92 | 0.03 (-0.07-0.12) | 0.58 |  | CAD | 4 (2 203) | 0.92 | 0.03 (-0.07-0.12) | 0.58 |
| Healthy subjects | 6 (4 668) | 0.28 | -0.00 (-0.05-0.05) | 0.90 |  | Healthy subjects | 6 (4 668) | 0.28 | -0.00 (-0.05-0.05) | 0.90 |
| **HDL-C** |  |  |  |  |  | **HDL-C** |  |  |  |  |
| All | 17 (21 090) | <0.001 | -0.02 (-0.11-0.07) | 0.70 |  | All | 14 (17 162) | 0.23 | -0.01 (-0.04-0.03) | 0.65 |
| Studies in HWE | 15 (20 178) | <0.001 | -0.01 (-0.11-0.08) | 0.77 |  | Studies in HWE | 12 (16 250) | 0.21 | -0.00 (-0.03-0.03) | 0.66 |
| Caucasian | 9 (13 258) | <0.001 | -0.01 (-0.15-0.14) | 0.91 |  | Caucasian | 7 (9 603) | 0.59 | -0.01 (-0.06-0.04) | 0.75 |
| Asian | 6 (7 052) | 0.09 | -0.02 (-0.10-0.07) | 0.71 |  | Asian | 6 (7 052) | 0.09 | -0.02 (-0.07-0.03) | 0.49 |
| Other ethnicity | 2 (780) | 0.01 | -0.09 (-0.58-0.40) | 0.71 |  | Other ethnicity | - | - | - | - |
| CAD | 4 (2 741) | 0.81 | 0.07 (-0.02-0.17) | 0.14 |  | CAD | 4 (2 741) | 0.81 | 0.07 (-0.02-0.17) | 0.14 |
| Healthy subjects | 7 (10 547) | 0.31 | 0.00 (-0.05-0.05) | 0.92 |  | Healthy subjects | 7 (10 547) | 0.31 | -0.00 (-0.04-0.04) | 0.99 |

*lncRNA*: long non-coding RNA; SMD: standardized mean difference; 95% CI: 95% confidence interval; HWE: Hardy-Weinberg equilibrium; CAD: coronary artery disease; TG: triglycerides; TC: total cholesterol; LDL-C: low-density lipoprotein cholesterol; HDL-C: high-density lipoprotein cholesterol.

**Table S8.** Meta-analysis of lncRNA rs4977574 variant with circulating lipid levels.

| **Groups or subgroups** | **Comparisons**  **(Subjects)** | ***P*_H_** | **SMD (95% CI)** | ***P*_SMD_** |  | **Groups or subgroups** | **Comparisons**  **(Subjects)** | ***P*_H_** | **SMD (95% CI)** | ***P*_SMD_** |
| --- | --- | --- | --- | --- | --- | --- | --- | --- | --- | --- |
| ***Overall results*** | | | | |  | ***Recalculated results that eliminated heterogeneity*** | | | | |
| **TG** |  |  |  |  |  | **TG** |  |  |  |  |
| All | 9 (30 846) | 0.01 | -0.02 (-0.10-0.06) | 0.70 |  | All | 7 (30 622) | 0.44 | -0.03 (-0.05--0.00) | 0.03 |
| Studies in HWE | 9 (30 846) | 0.01 | -0.02 (-0.10-0.06) | 0.70 |  | Studies in HWE | 7 (30 622) | 0.44 | -0.03 (-0.05--0.00) | 0.03 |
| Caucasian | 4 (24 912) | 0.75 | -0.03 (-0.06--0.00) | 0.04 |  | Caucasian | 4 (24 912) | 0.75 | -0.03 (-0.06--0.00) | 0.04 |
| Asian | 3 (5 710) | 0.10 | -0.05 (-0.16-0.06) | 0.38 |  | Asian | 3 (5 710) | 0.10 | -0.02 (-0.08-0.04) | 0.50 |
| CAD | 3 (1 096) | 0.01 | 0.16 (-0.33-0.66) | 0.52 |  | CAD | 2 (1 025) | 0.48 | -0.04 (-0.19-0.11) | 0.59 |
| Healthy subjects | 3 (24 195) | 0.05 | 0.07 (-0.24-0.37) | 0.67 |  | Healthy subjects | 2 (24 042) | 0.51 | -0.03 (-0.05-0.00) | 0.05 |
| **TC** |  |  |  |  |  | **TC** |  |  |  |  |
| All | 7 (2 791) | 0.88 | -0.06 (-0.15-0.02) | 0.13 |  | All | 7 (2 791) | 0.88 | -0.06 (-0.15-0.02) | 0.13 |
| Studies in HWE | 7 (2 791) | 0.88 | -0.06 (-0.15-0.02) | 0.13 |  | Studies in HWE | 7 (2 791) | 0.88 | -0.06 (-0.15-0.02) | 0.13 |
| Caucasian | 3 (963) | 0.57 | -0.02 (-0.16-0.11) | 0.73 |  | Caucasian | 3 (963) | 0.57 | -0.02 (-0.16-0.11) | 0.73 |
| Asian | 2 (1 604) | 0.72 | -0.11 (-0.22-0.01) | 0.07 |  | Asian | 2 (1 604) | 0.72 | -0.11 (-0.22-0.01) | 0.07 |
| CAD | 3 (1 096) | 0.53 | -0.05 (-0.20-0.09) | 0.48 |  | CAD | 3 (1 096) | 0.53 | -0.05 (-0.20-0.09) | 0.48 |
| Healthy subjects | 2 (246) | 0.81 | 0.04 (-0.26-0.34) | 0.79 |  | Healthy subjects | 2 (246) | 0.81 | 0.04 (-0.26-0.34) | 0.79 |
| **LDL-C** |  |  |  |  |  | **LDL-C** |  |  |  |  |
| All | 11 (31 762) | 0.01 | 0.01 (-0.06-0.08) | 0.73 |  | All | 10 (31 691) | 0.15 | 0.02 (0.00-0.05) | 0.05 |
| Studies in HWE | 11 (31 762) | 0.01 | 0.01 (-0.06-0.08) | 0.73 |  | Studies in HWE | 10 (31 691) | 0.15 | 0.02 (0.00-0.05) | 0.05 |
| Caucasian | 4 (24 912) | 0.49 | 0.04 (0.01-0.06) | 0.01 |  | Caucasian | 4 (24 912) | 0.49 | 0.04 (0.01-0.06) | 0.01 |
| Asian | 5 (6 626) | 0.25 | -0.03 (-0.10-0.05) | 0.48 |  | Asian | 5 (6 626) | 0.25 | -0.03 (-0.09-0.02) | 0.25 |
| CAD | 4 (1 385) | <0.01 | 0.18 (-0.15-0.51) | 0.28 |  | CAD | 3 (1 314) | 0.19 | -0.04 (-0.17-0.08) | 0.49 |
| Healthy subjects | 3 (24 195) | 0.85 | 0.04 (0.01-0.07) | <0.01 |  | Healthy subjects | 3 (24 195) | 0.85 | 0.04 (0.01-0.07) | <0.01 |
| **HDL-C** |  |  |  |  |  | **HDL-C** |  |  |  |  |
| All | 9 (30 846) | 0.01 | 0.04 (-0.04-0.11) | 0.34 |  | All | 8 (29 921) | 0.10 | 0.02 (-0.01-0.04) | 0.18 |
| Studies in HWE | 9 (30 846) | 0.24 | -0.00 (-0.04-0.04) | 0.87 |  | Studies in HWE | 8 (29 921) | 0.10 | 0.02 (-0.01-0.04) | 0.18 |
| Caucasian | 4 (24 912) | 0.44 | 0.03 (0.00-0.06) | 0.04 |  | Caucasian | 4 (24 912) | 0.44 | 0.03 (0.00-0.06) | 0.04 |
| Asian | 3 (5 710) | 0.01 | 0.05 (-0.12-0.22) | 0.55 |  | Asian | 2 (4 785) | 0.59 | -0.04 (-0.11-0.02) | 0.19 |
| CAD | 3 (1 096) | 0.02 | 0.00 (-0.43-0.43) | 1.00 |  | CAD | 2 (171) | 0.06 | -0.16 (-0.51-0.18) | 0.35 |
| Healthy subjects | 3 (24 195) | 0.21 | 0.10 (-0.09-0.29) | 0.29 |  | Healthy subjects | 3 (24 195) | 0.21 | 0.03 (0.00-0.06) | 0.04 |

*lncRNA*: long non-coding RNA; SMD: standardized mean difference; 95% CI: 95% confidence interval; HWE: Hardy-Weinberg equilibrium; CAD: coronary artery disease; TG: triglycerides; TC: total cholesterol; LDL-C: low-density lipoprotein cholesterol; HDL-C: high-density lipoprotein cholesterol.

**Table S9.** Meta-analysis of lncRNA rs10757274 variant with circulating lipid levels.

| **Groups or subgroups** | **Comparisons**  **(Subjects)** | ***P*_H_** | **SMD (95% CI)** | ***P*_SMD_** |  | **Groups or subgroups** | **Comparisons**  **(Subjects)** | ***P*_H_** | **SMD (95% CI)** | ***P*_SMD_** |
| --- | --- | --- | --- | --- | --- | --- | --- | --- | --- | --- |
| ***Overall results*** | | | | |  | ***Recalculated results that eliminated heterogeneity*** | | | | |
| **TG** |  |  |  |  |  | **TG** |  |  |  |  |
| All | 6 (20 887) | <0.001 | -0.04 (-0.22-0.13) | 0.62 |  | All | 4 (20 457) | 0.89 | -0.03 (-0.06--0.00) | 0.05 |
| Studies in HWE | 6 (20 887) | <0.001 | -0.04 (-0.22-0.13) | 0.62 |  | Studies in HWE | 4 (20 457) | 0.89 | -0.03 (-0.06--0.00) | 0.05 |
| Caucasian | 2 (19 336) | 0.44 | -0.03 (-0.06-0.00) | 0.05 |  | Caucasian | 2 (19 336) | 0.44 | -0.03 (-0.06--0.00) | 0.05 |
| Asian | 2 (430) | <0.001 | -0.61 (-4.02-2.81) | 0.73 |  | Asian | - | - | - | - |
| Healthy subjects | 2 (19 336) | 0.44 | -0.03 (-0.06-0.00) | 0.05 |  | Healthy subjects | 2 (19 336) | 0.44 | -0.03 (-0.06--0.00) | 0.05 |
| **TC** |  |  |  |  |  | **TC** |  |  |  |  |
| All | 9 (36 636) | <0.001 | 0.04 (-0.11-0.18) | 0.63 |  | All | 7 (36 206) | 0.85 | -0.01 (-0.03-0.02) | 0.58 |
| Studies in HWE | 9 (36 636) | 0.88 | -0.06 (-0.15-0.02) | 0.13 |  | Studies in HWE | 7 (36 206) | 0.85 | -0.01 (-0.03-0.02) | 0.58 |
| Caucasian | 3 (30 421) | 0.94 | -0.01 (-0.03-0.02) | 0.62 |  | Caucasian | 3 (30 421) | 0.94 | -0.01 (-0.03-0.02) | 0.62 |
| Asian | 2 (430) | <0.001 | -0.80 (-6.93-5.32) | 0.80 |  | Asian | - | - | - | - |
| Other ethnicity | 4 (5 785) | 0.46 | -0.01 (-0.06-0.05) | 0.81 |  | Other ethnicity | 4 (5 785) | 0.46 | -0.01 (-0.06-0.05) | 0.81 |
| Healthy subjects | 4 (34 439) | 0.94 | -0.01 (-0.03-0.02) | 0.45 |  | Healthy subjects | 4 (34 439) | 0.94 | -0.01 (-0.03-0.02) | 0.45 |
| **LDL-C** |  |  |  |  |  | **LDL-C** |  |  |  |  |
| All | 7 (21 533) | <0.001 | 0.01 (-0.12-0.14) | 0.87 |  | All | 5 (21 103) | 0.76 | -0.01 (-0.04-0.02) | 0.62 |
| Studies in HWE | 63 (51 893) | <0.001 | 0.01 (-0.12-0.14) | 0.87 |  | Studies in HWE | 5 (21 103) | 0.76 | -0.01 (-0.04-0.02) | 0.62 |
| Caucasian | 2 (19 336) | 0.99 | -0.01 (-0.04-0.02) | 0.53 |  | Caucasian | 2 (19 336) | 0.99 | -0.01 (-0.04-0.02) | 0.53 |
| Asian | 2 (430) | <0.001 | -0.41 (-3.09-2.26) | 0.76 |  | Asian | - | - | - | - |
| Other ethnicity | 3 (1 767) | 0.47 | 0.03 (-0.09-0.14) | 0.67 |  | Other ethnicity | 3 (1 767) | 0.47 | 0.03 (-0.09-0.14) | 0.67 |
| Healthy subjects | 2 (19 336) | 0.99 | -0.01 (-0.04-0.02) | 0.53 |  | Healthy subjects | 2 (19 336) | 0.99 | -0.01 (-0.04-0.02) | 0.53 |
| **HDL-C** |  |  |  |  |  | **HDL-C** |  |  |  |  |
| All | 9 (36 636) | <0.001 | 0.22 (0.04-0.39) | 0.01 |  | All | 7 (36 206) | 0.10 | 0.03 (0.01-0.05) | 0.01 |
| Studies in HWE | 9 (36 636) | <0.001 | 0.22 (0.04-0.39) | 0.01 |  | Studies in HWE | 7 (36 206) | 0.10 | 0.03 (0.01-0.06) | 0.01 |
| Caucasian | 3 (30 421) | 1.00 | 0.05 (0.02-0.07) | <0.001 |  | Caucasian | 3 (30 421) | 1.00 | 0.05 (0.02-0.07) | <0.001 |
| Asian | 2 (430) | <0.001 | 2.14 (-4.35-8.63) | 0.52 |  | Asian | - | - | - | - |
| Other ethnicity | 4 (5 785) | 0.35 | -0.04 (-0.10-0.03) | 0.26 |  | Other ethnicity | 4 (5 785) | 0.36 | -0.04 (-0.10-0.02) | 0.17 |
| Healthy subjects | 4 (34 439) | 0.09 | 0.03 (-0.01-0.07) | 0.11 |  | Healthy subjects | 4 (34 439) | 0.09 | 0.03 (0.01-0.06) | 0.01 |

*lncRNA*: long non-coding RNA; SMD: standardized mean difference; 95% CI: 95% confidence interval; HWE: Hardy-Weinberg equilibrium; TG: triglycerides; TC: total cholesterol; LDL-C: low-density lipoprotein cholesterol; HDL-C: high-density lipoprotein cholesterol.

**Table S10.** Meta-analysis of lncRNA rs10757278 variant with circulating lipid levels.

| **Groups or subgroups** | **Comparisons**  **(Subjects)** | ***P*_H_** | **SMD (95% CI)** | ***P*_SMD_** |  | **Groups or subgroups** | **Comparisons**  **(Subjects)** | ***P*_H_** | **SMD (95% CI)** | ***P*_SMD_** |
| --- | --- | --- | --- | --- | --- | --- | --- | --- | --- | --- |
| ***Overall results*** | | | | |  | ***Recalculated results that eliminated heterogeneity*** | | | | |
| **TG** |  |  |  |  |  | **TG** |  |  |  |  |
| All | 4 (2 672) | 0.99 | -0.00 (-0.09-0.08) | 0.92 |  | All | 4 (2 672) | 0.99 | -0.00 (-0.09-0.08) | 0.92 |
| Studies in HWE | 4 (2 672) | 0.99 | -0.00 (-0.09-0.09) | 0.98 |  | Studies in HWE | 4 (2 672) | 0.99 | -0.00 (-0.09-0.09) | 0.98 |
| CAD | 2 (933) | 0.96 | -0.00 (-0.15-0.15) | 0.96 |  | CAD | 2 (933) | 0.96 | -0.00 (-0.15-0.15) | 0.96 |
| **TC** |  |  |  |  |  | **TC** |  |  |  |  |
| All | 7 (3 533) | <0.01 | 0.13 (-0.10-0.35) | 0.26 |  | All | 6 (3 483) | 0.28 | 0.02 (-0.06-0.09) | 0.68 |
| Studies in HWE | 7 (3 533) | <0.01 | 0.13 (-0.10-0.35) | 0.26 |  | Studies in HWE | 6 (3 483) | 0.28 | 0.02 (-0.06-0.09) | 0.68 |
| Caucasian | 5 (2 921) | <0.01 | 0.11 (-0.22-0.44) | 0.53 |  | Caucasian | 4 (2 871) | 0.65 | -0.02 (-0.10-0.07) | 0.71 |
| CAD | 5 (3 367) | <0.01 | 0.11 (-0.16-0.37) | 0.42 |  | CAD | 4 (3 317) | 0.45 | 0.00 (-0.08-0.08) | 0.96 |
| **LDL-C** |  |  |  |  |  | **LDL-C** |  |  |  |  |
| All | 5 (5 006) | 0.20 | 0.02 (-0.05-0.08) | 0.63 |  | All | 4 (4 890) | 0.76 | 0.00 (-0.06-0.07) | 0.91 |
| Studies in HWE | 5 (5 006) | 0.20 | 0.02 (-0.05-0.08) | 0.63 |  | Studies in HWE | 4 (4 890) | 0.76 | 0.00 (-0.06-0.07) | 0.91 |
| Caucasian | 2 (2 771) | 0.72 | -0.02 (-0.11-0.06) | 0.56 |  | Caucasian | 2 (2 771) | 0.72 | -0.02 (-0.11-0.06) | 0.56 |
| Other ethnicity | 2 (2 119) | 0.82 | 0.04 (-0.06-0.14) | 0.42 |  | Other ethnicity | 2 (2 119) | 0.82 | 0.04 (-0.06-0.14) | 0.42 |
| CAD | 3 (3 267) | 0.70 | -0.01 (-0.09-0.07) | 0.75 |  | CAD | 3 (3 267) | 0.70 | -0.01 (-0.09-0.07) | 0.75 |
| **HDL-C** |  |  |  |  |  | **HDL-C** |  |  |  |  |
| All | 4 (4 890) | 0.54 | -0.01 (-0.07-0.06) | 0.79 |  | All | 4 (4 890) | 0.54 | -0.01 (-0.07-0.06) | 0.79 |
| Studies in HWE | 4 (4 890) | 0.54 | -0.01 (-0.07-0.06) | 0.79 |  | Studies in HWE | 4 (4 890) | 0.54 | -0.01 (-0.07-0.06) | 0.79 |
| Caucasian | 2 (2 771) | 0.94 | 0.00 (-0.09-0.09) | 0.98 |  | Caucasian | 2 (2 771) | 0.94 | 0.00 (-0.09-0.09) | 0.98 |
| Other ethnicity | 2 (2 119) | 0.15 | -0.02 (-0.12-0.08) | 0.66 |  | Other ethnicity | 2 (2 119) | 0.15 | -0.02 (-0.12-0.08) | 0.66 |
| CAD | 3 (3 267) | 0.62 | 0.02 (-0.06-0.10) | 0.66 |  | CAD | 3 (3 267) | 0.62 | 0.02 (-0.06-0.10) | 0.66 |

*lncRNA*: long non-coding RNA; SMD: standardized mean difference; 95% CI: 95% confidence interval; HWE: Hardy-Weinberg equilibrium; CAD: coronary artery disease; TG: triglycerides; TC: total cholesterol; LDL-C: low-density lipoprotein cholesterol; HDL-C: high-density lipoprotein cholesterol.

**Table S11.** Meta-analysis of CDKN2A/2B rs10811661 variant with circulating lipid levels.

| **Groups or subgroups** | **Comparisons**  **(Subjects)** | ***P*_H_** | **SMD (95% CI)** | ***P*_SMD_** |  | **Groups or subgroups** | **Comparisons**  **(Subjects)** | ***P*_H_** | **SMD (95% CI)** | ***P*_SMD_** |
| --- | --- | --- | --- | --- | --- | --- | --- | --- | --- | --- |
| ***Overall results*** | | | | |  | ***Recalculated results that eliminated heterogeneity*** | | | | |
| **TG** |  |  |  |  |  | **TG** |  |  |  |  |
| All | 9 (5 402) | <0.01 | -0.14 (-0.26--0.02) | 0.02 |  | All | 7 (4 960) | 0.14 | -0.02 (-0.08-0.04) | 0.43 |
| Studies in HWE | 9 (5 402) | <0.01 | -0.14 (-0.26--0.02) | 0.02 |  | Studies in HWE | 7 (4 960) | 0.14 | -0.02 (-0.08-0.04) | 0.43 |
| Caucasian | 3 (964) | 0.64 | -0.24 (-0.38--0.10) | <0.01 |  | Caucasian | 2 (642) | 0.94 | -0.19 (-0.37--0.01) | 0.04 |
| Asian | 5 (3 281) | <0.01 | -0.15 (-0.33-0.04) | 0.11 |  | Asian | 4 (3 161) | 0.16 | -0.02 (-0.10-0.06) | 0.58 |
| T2DM | 3 (2 024) | 0.22 | -0.07 (-0.21-0.06) | 0.28 |  | T2DM | 3 (2 024) | 0.22 | -0.06 (-0.16-0.03) | 0.20 |
| Healthy subjects | 2 (1 534) | 0.07 | -0.04 (-0.28-0.19) | 0.71 |  | Healthy subjects | 2 (1 534) | 0.07 | -0.00 (-0.11-0.11) | 0.97 |
| **TC** |  |  |  |  |  | **TC** |  |  |  |  |
| All | 9 (3 726) | 0.28 | -0.07 (-0.14--0.00) | 0.05 |  | All | 9 (3 726) | 0.28 | -0.07 (-0.14--0.00) | 0.05 |
| Studies in HWE | 9 (3 726) | 0.28 | -0.07 (-0.14--0.00) | 0.05 |  | Studies in HWE | 9 (3 726) | 0.28 | -0.07 (-0.14--0.00) | 0.05 |
| Caucasian | 3 (964) | 0.47 | -0.01 (-0.15-0.14) | 0.93 |  | Caucasian | 3 (964) | 0.47 | -0.01 (-0.15-0.14) | 0.93 |
| Asian | 3 (979) | 0.20 | -0.14 (-0.28--0.00) | 0.05 |  | Asian | 3 (979) | 0.20 | -0.14 (-0.28--0.00) | 0.05 |
| Other ethnicity | 3 (1 783) | 0.19 | -0.06 (-0.16-0.03) | 0.20 |  | Other ethnicity | 3 (1 783) | 0.19 | -0.06 (-0.16-0.03) | 0.20 |
| T2DM | 4 (1 485) | 0.17 | -0.15 (-0.26--0.04) | 0.01 |  | T2DM | 4 (1 485) | 0.17 | -0.15 (-0.26--0.04) | 0.01 |
| **LDL-C** |  |  |  |  |  | **LDL-C** |  |  |  |  |
| All | 8 (3 226) | <0.001 | -0.22 (-0.45-0.02) | 0.07 |  | All | 4 (756) | 0.06 | -0.63 (-0.81--0.46) | <0.001 |
| Studies in HWE | 8 (3 226) | <0.001 | -0.22 (-0.45-0.02) | 0.07 |  | Studies in HWE | 4 (756) | 0.06 | -0.63 (-0.81--0.46) | <0.001 |
| Caucasian | 3 (964) | <0.001 | -0.26 (-0.87-0.37) | 0.42 |  | Caucasian | - | - | - | - |
| Asian | 3 (979) | 0.17 | -0.15 (-0.39-0.10) | 0.25 |  | Asian | 2 (233) | 0.48 | -0.32 (-0.62--0.02) | 0.04 |
| Other ethnicity | 2 (1 283) | 0.05 | -0.23 (-0.76-0.31) | 0.41 |  | Other ethnicity | - | - | - | - |
| T2DM | 3 (985) | 0.15 | -0.17 (-0.48-0.14) | 0.29 |  | T2DM | 2 (239) | 0.29 | -0.38 (-0.76-0.00) | 0.05 |
| **HDL-C** |  |  |  |  |  | **HDL-C** |  |  |  |  |
| All | 8 (3 226) | 0.01 | 0.03 (-0.11-0.18) | 0.66 |  | All | 7 (2 829) | 0.12 | -0.02 (-0.10-0.06) | 0.65 |
| Studies in HWE | 8 (3 226) | 0.01 | 0.03 (-0.11-0.18) | 0.66 |  | Studies in HWE | 7 (2 829) | 0.12 | -0.02 (-0.10-0.06) | 0.65 |
| Caucasian | 3 (964) | 0.02 | 0.09 (-0.20-0.38) | 0.55 |  | Caucasian | 2 (567) | 0.19 | -0.03 (-0.22-0.15) | 0.73 |
| Asian | 3 (979) | 0.07 | 0.07 (-0.24-0.40) | 0.63 |  | Asian | 3 (979) | 0.07 | -0.00 (-0.14-0.13) | 0.95 |
| Other ethnicity | 2 (1 283) | 0.08 | -0.18 (-0.65-0.29) | 0.46 |  | Other ethnicity | 2 (1 283) | 0.08 | -0.02 (-0.13-0.09) | 0.71 |
| T2DM | 3 (985) | 0.33 | -0.12 (-0.30-0.07) | 0.21 |  | T2DM | 3 (985) | 0.33 | -0.10 (-0.25-0.04) | 0.17 |

*CDKN2A/2B*: cyclin-dependent kinase inhibitor 2A/2B; SMD: standardized mean difference; 95% CI: 95% confidence interval; HWE: Hardy-Weinberg equilibrium; T2DM: type 2 diabetes mellitus; TG: triglycerides; TC: total cholesterol; LDL-C: low-density lipoprotein cholesterol; HDL-C: high-density lipoprotein cholesterol.


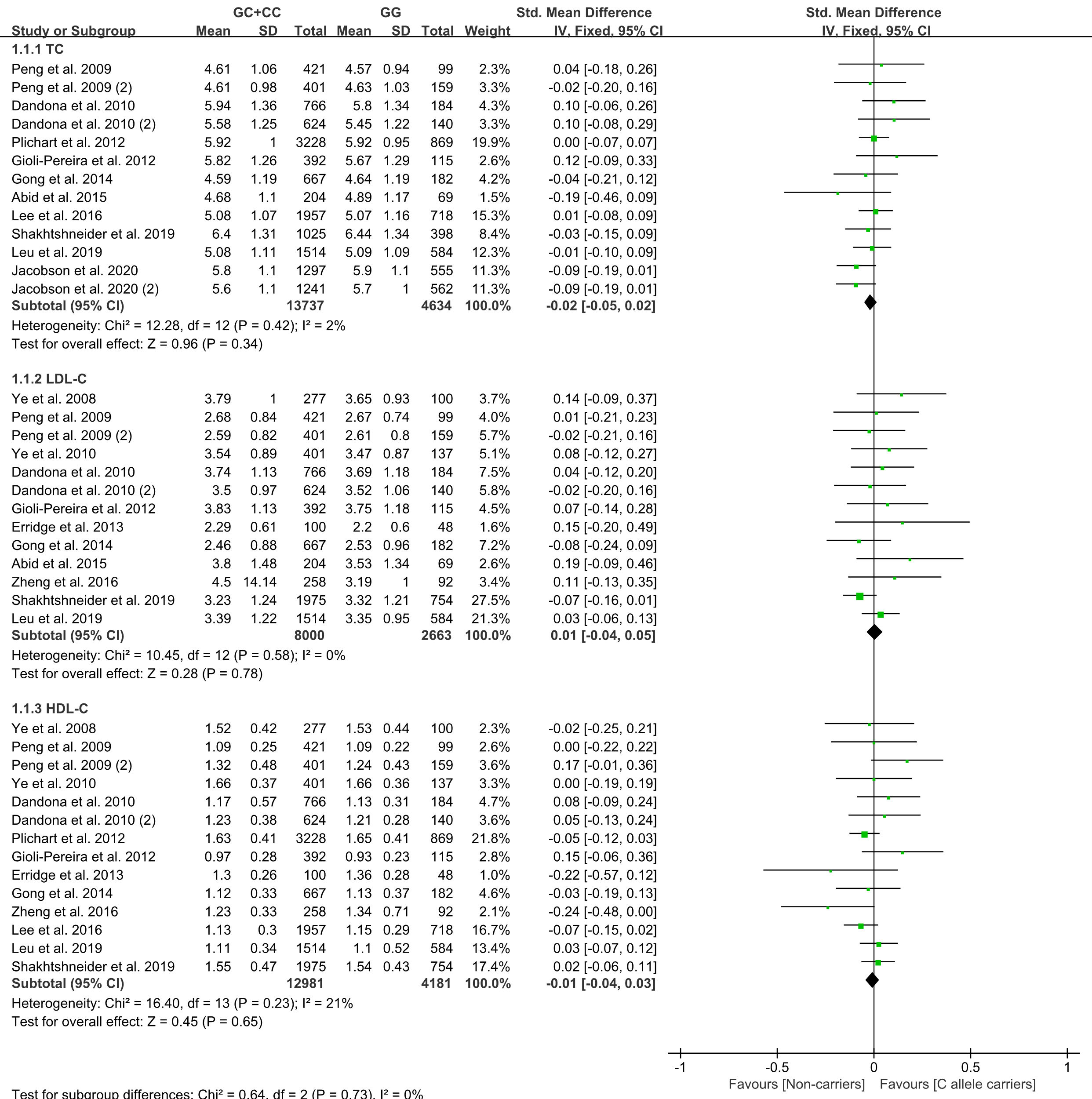


**Figure S1.** Forest plot of lncRNA rs1333049 variant with circulating TC, LDL-C and HDL-C levels.


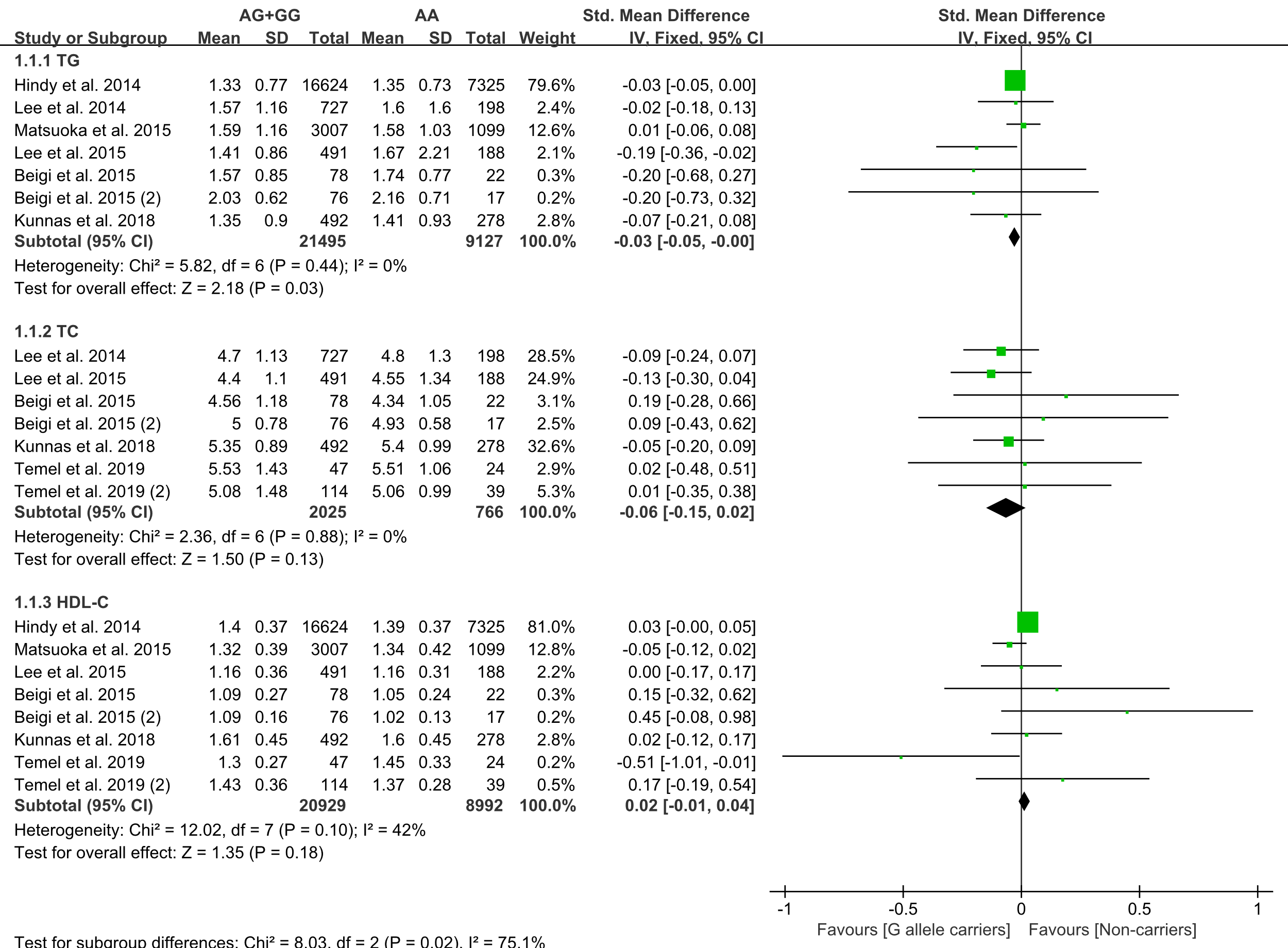


**Figure S2.** Forest plot of lncRNA rs4977574 variant with circulating TG, TC and HDL-C levels.


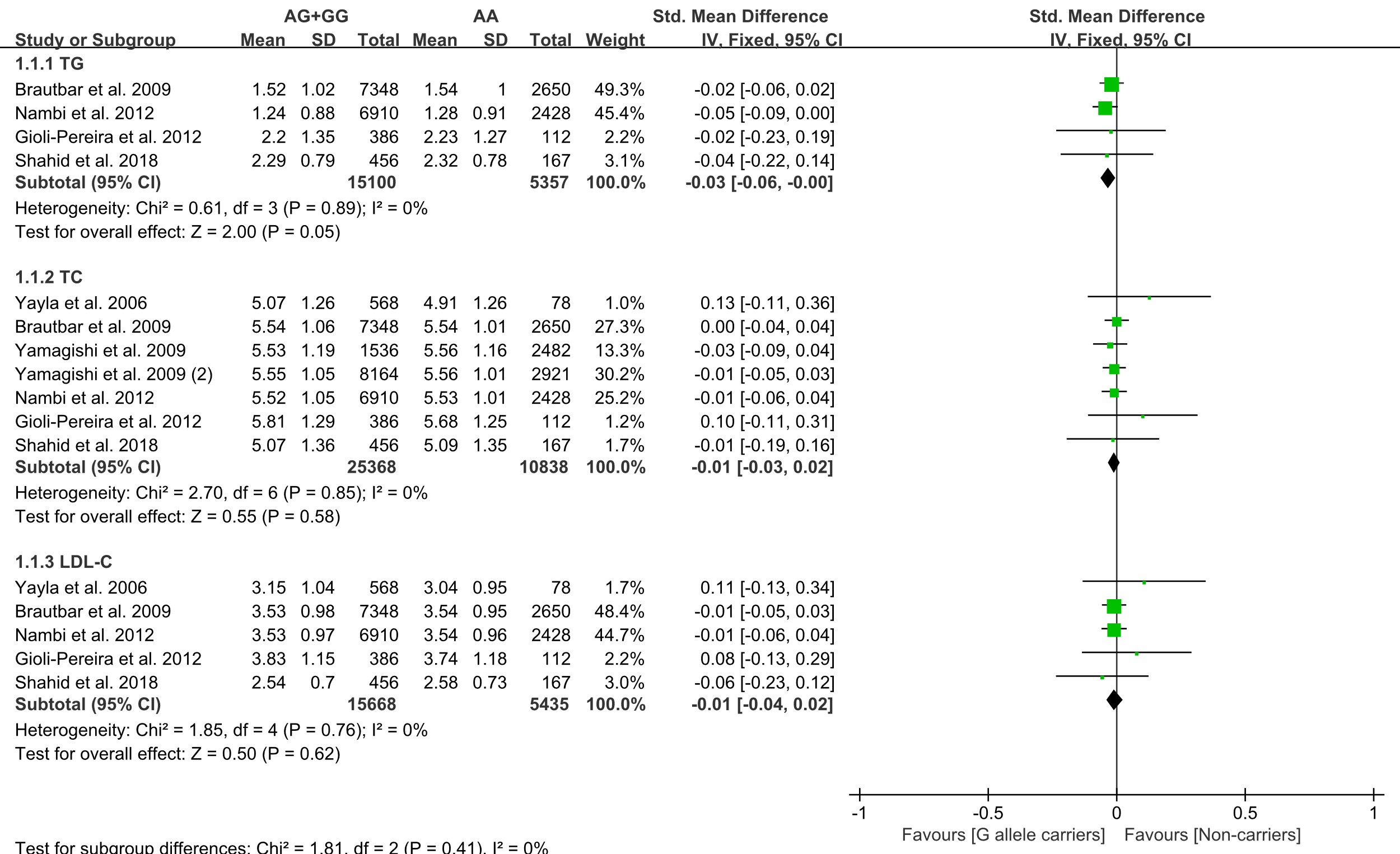


**Figure S3.**Forest plot of lncRNA rs10757274 variant with circulating TG, TC and LDL-C levels.


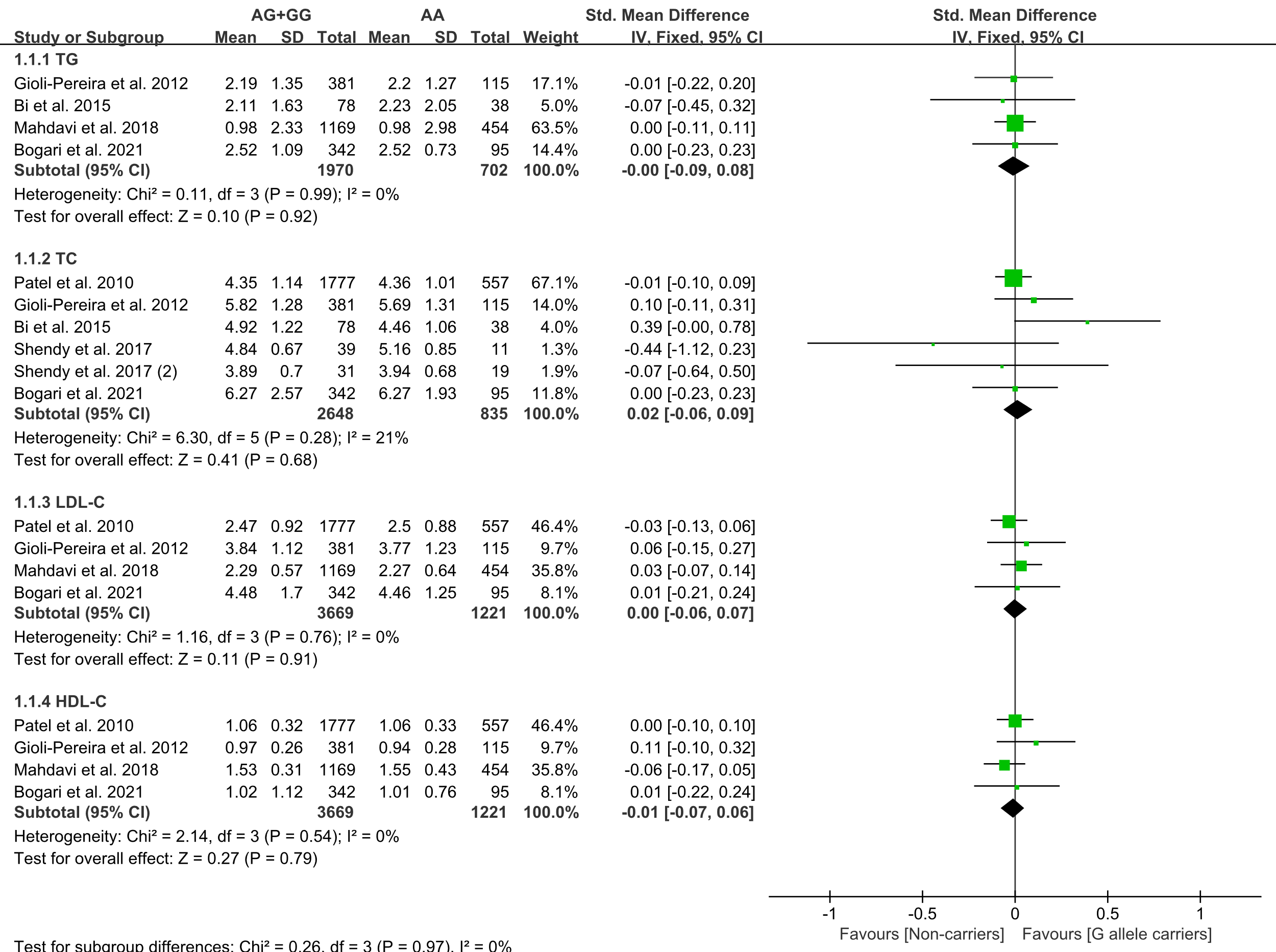


**Figure S4.** Forest plot of lncRNA rs10757278 variant with circulating lipid levels.


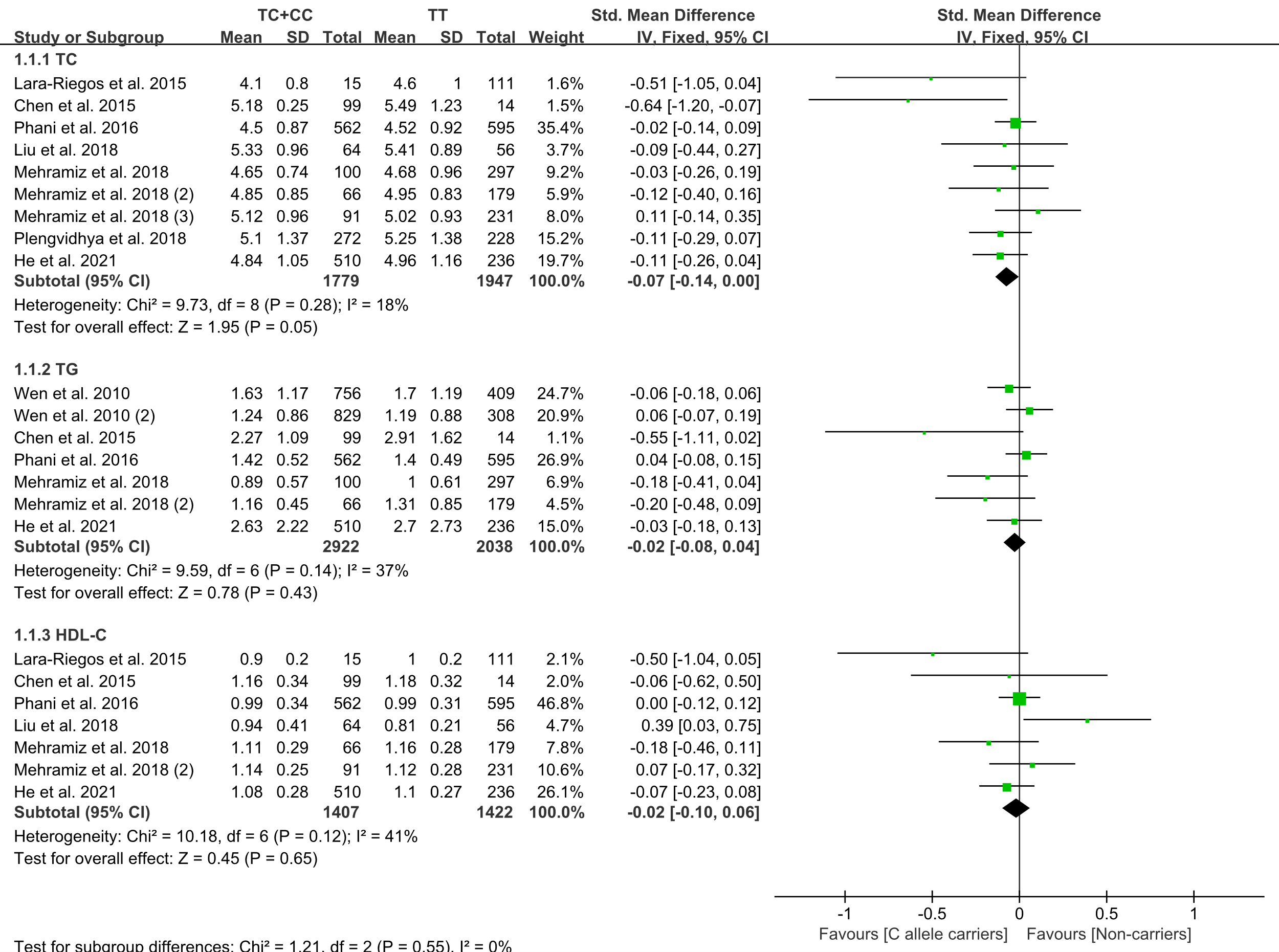


**Figure S5.** Forest plot of CDKN2A/2B rs10811661 variant with circulating TC, TG and HDL-C levels.


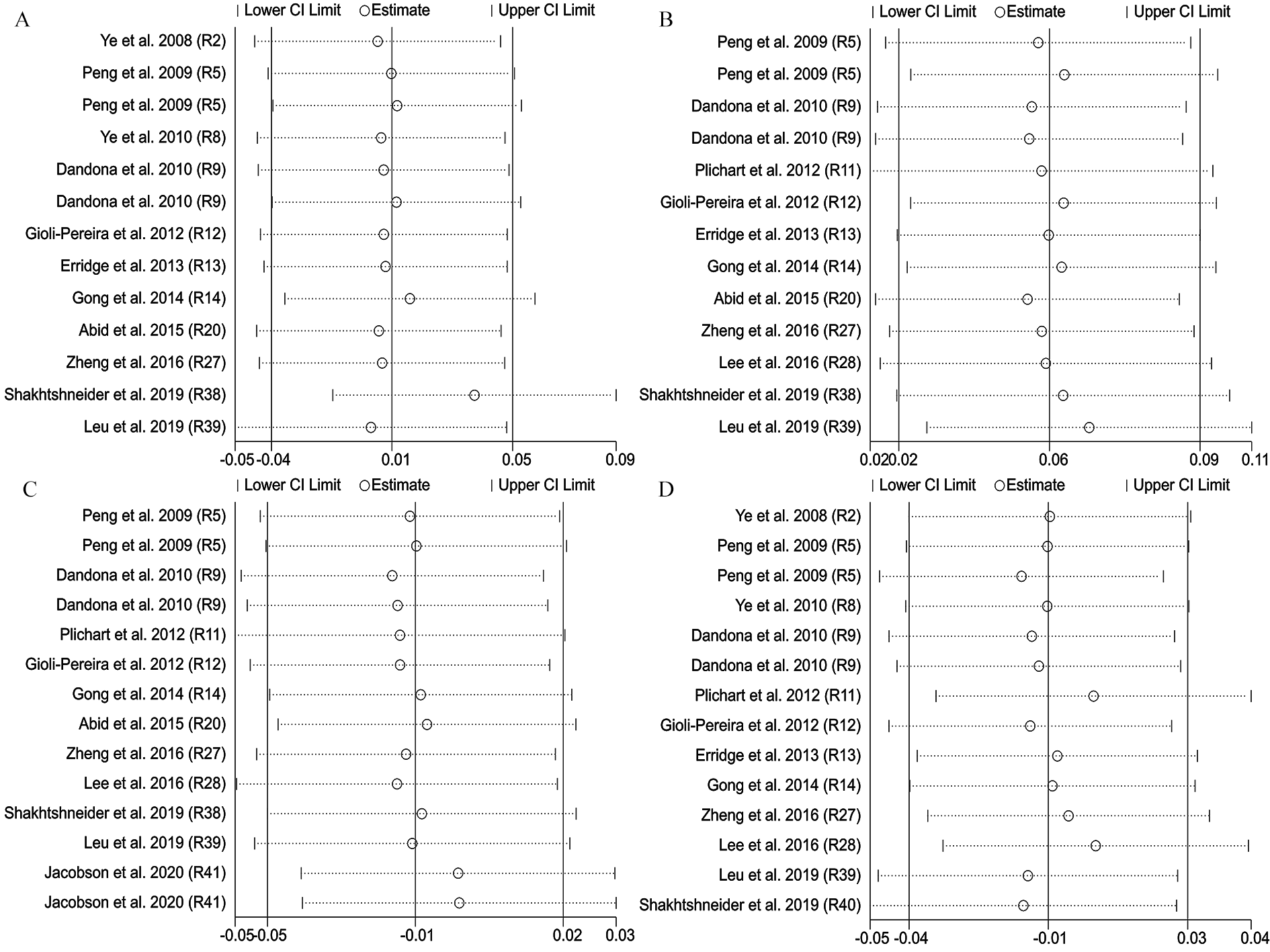


**Figure S6.** Sensitivity analysis between lncRNA rs1333049 variant and circulating lipid levels. Open circle is SMD, parallel lines

represent 95% CI (A: rs1333049 on LDL-C levels; B: rs1333049 on TG levels; C: rs1333049 on TC levels; D: rs1333049 on HDL-C levels).


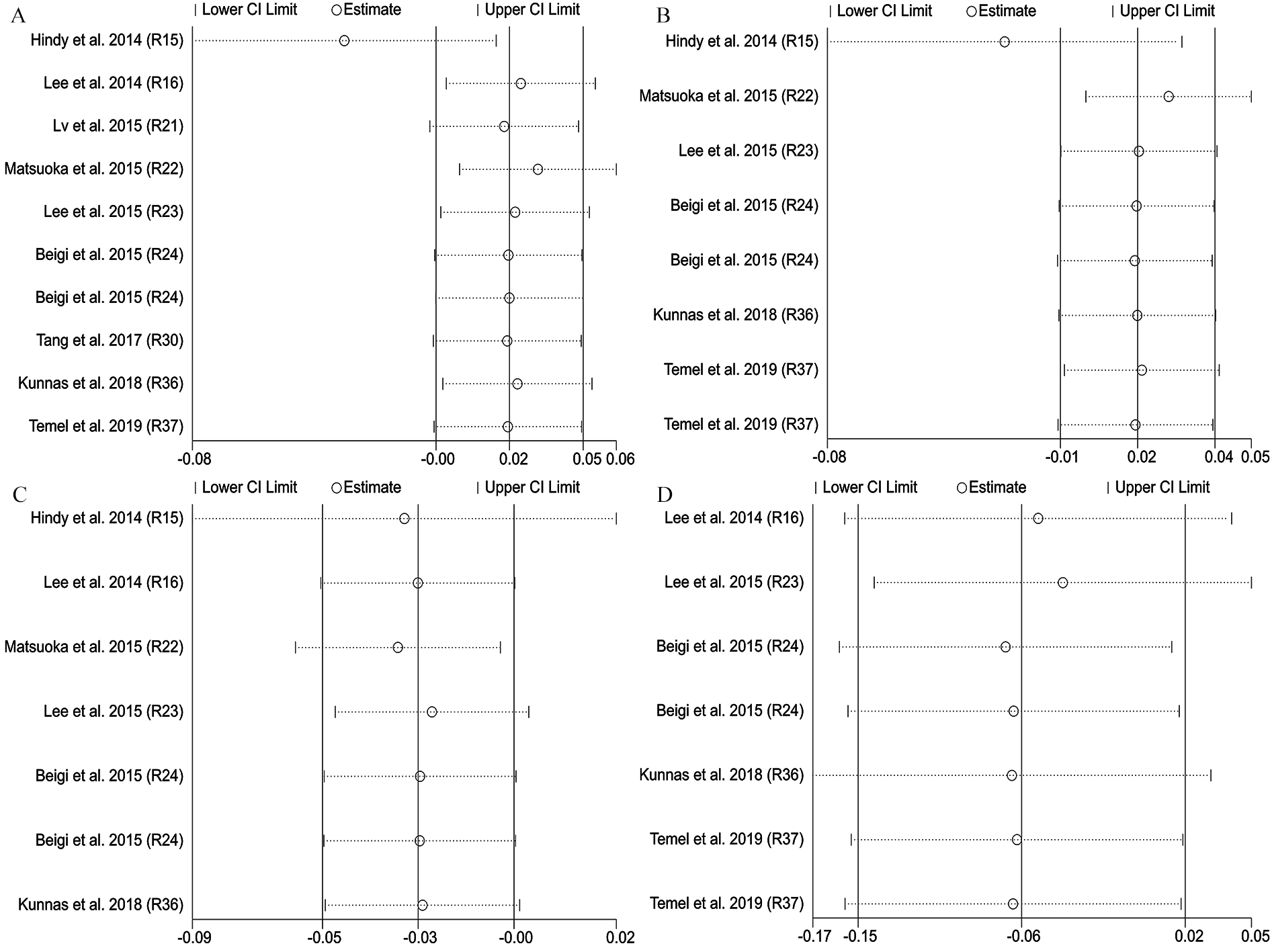


**Figure S7.** Sensitivity analysis between lncRNA rs4977574 variant and circulating lipid levels. Open circle is SMD, parallel lines represent 95% CI (A: rs4977574 on LDL-C levels; B: rs4977574 on HDL-C levels; C: rs4977574 on TG levels; D: rs4977574 on TC levels).


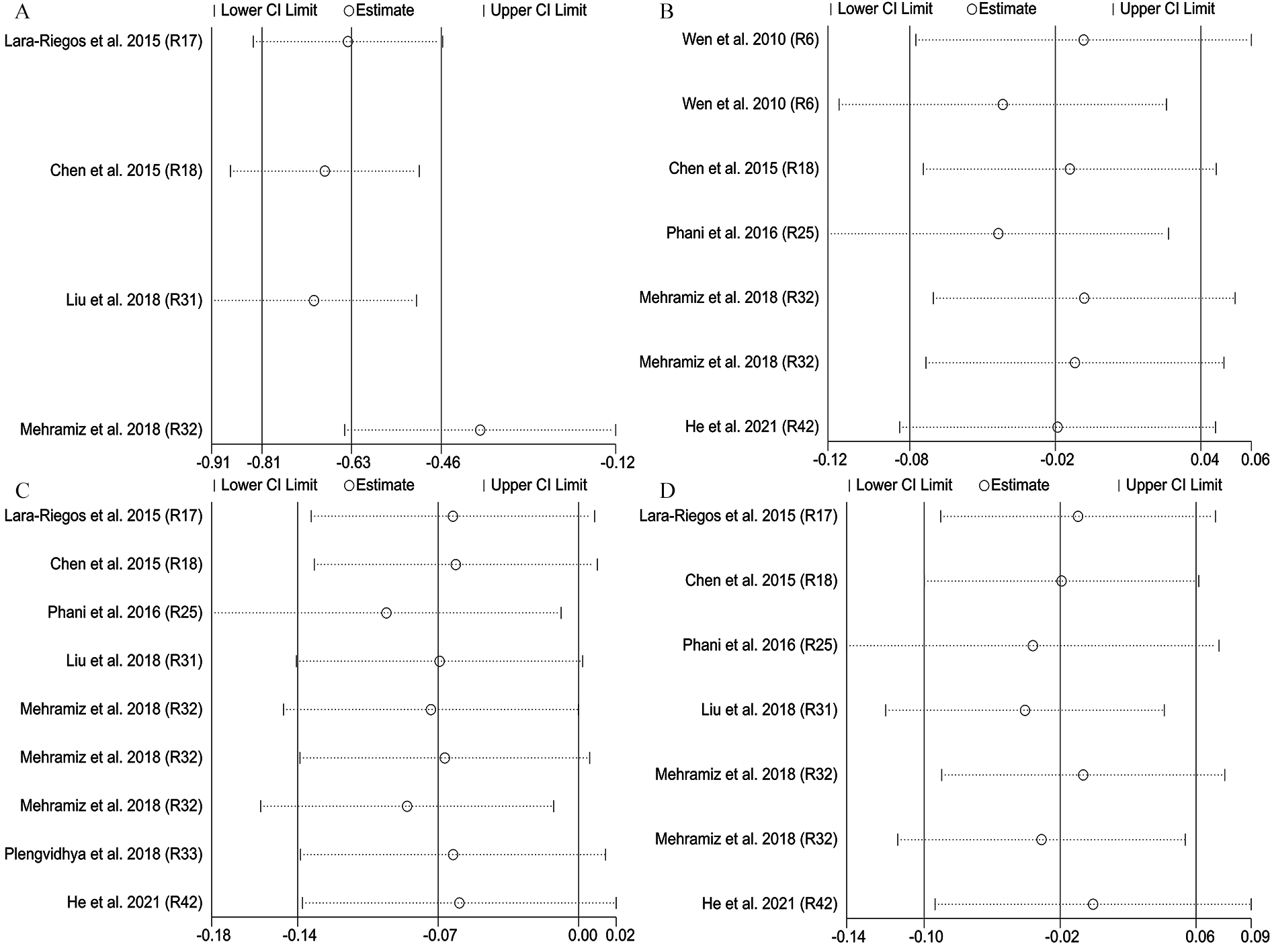


**Figure S8.** Sensitivity analysis between CDKN2A/2B rs10811661 variant and circulating lipid levels. Open circle is SMD, parallel lines represent 95% CI (A: rs10811661 on LDL-C levels; B: rs10811661 on TG levels; C: rs10811661 on TC levels; D: rs10811661 on HDL-C levels).


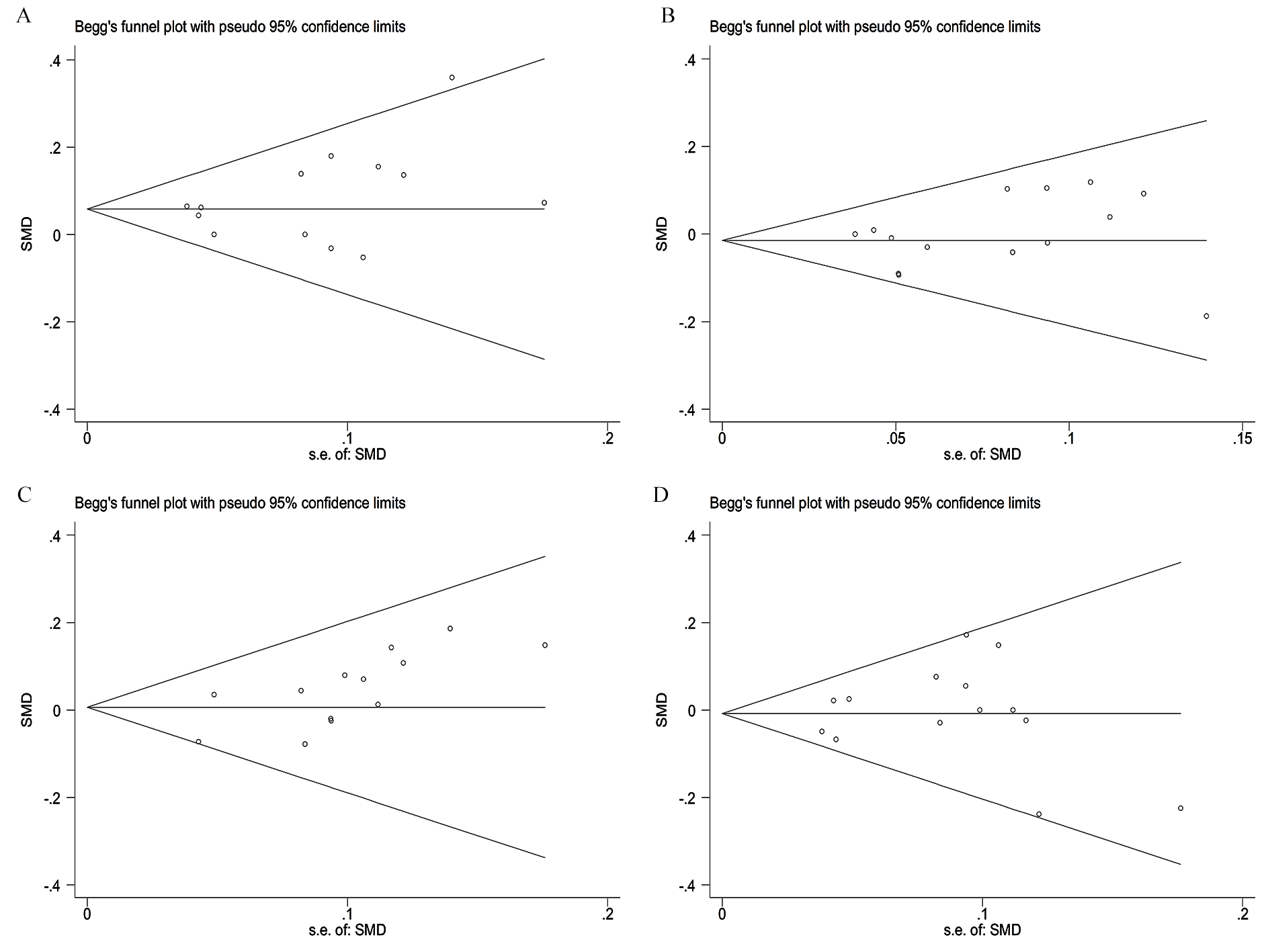


**Figure S9.** Begg’s funnel plot of the effects of lncRNA rs1333049 variant and circulating lipid levels. Each small circle represents a separate study, the diverging lines represent 95% CI and the central line is SMD. [A: rs1333049 on TG levels (*P* = 0.81); B: rs1333049 on TC levels (*P* = 0.26); C: rs1333049 on LDL-C levels (*P* = 0.06); D: rs1333049 on HDL-C levels (*P* = 0.63)]


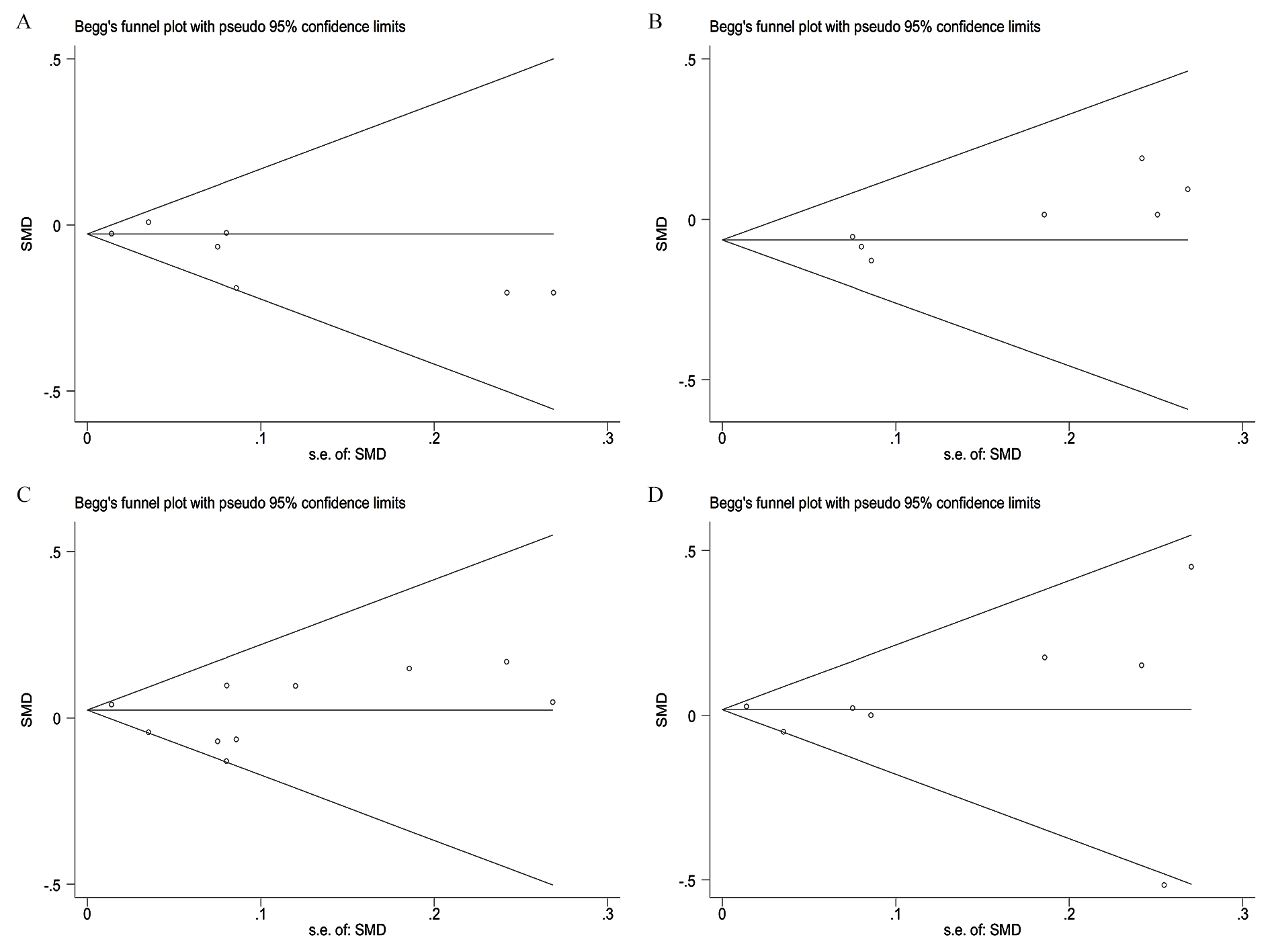


**Figure S10.** Begg’s funnel plot of the effects of lncRNA rs4977574 variant and circulating lipid levels. Each small circle represents a separate study, the diverging lines represent 95% CI and the central line is SMD. [A: rs4977574 on TG levels (*P* = 0.50); B: rs4977574 on TC levels (*P* = 0.18); C: rs4977574 on LDL-C levels (*P* = 0.14); D: rs4977574 on HDL-C levels (*P* = 0.45)]


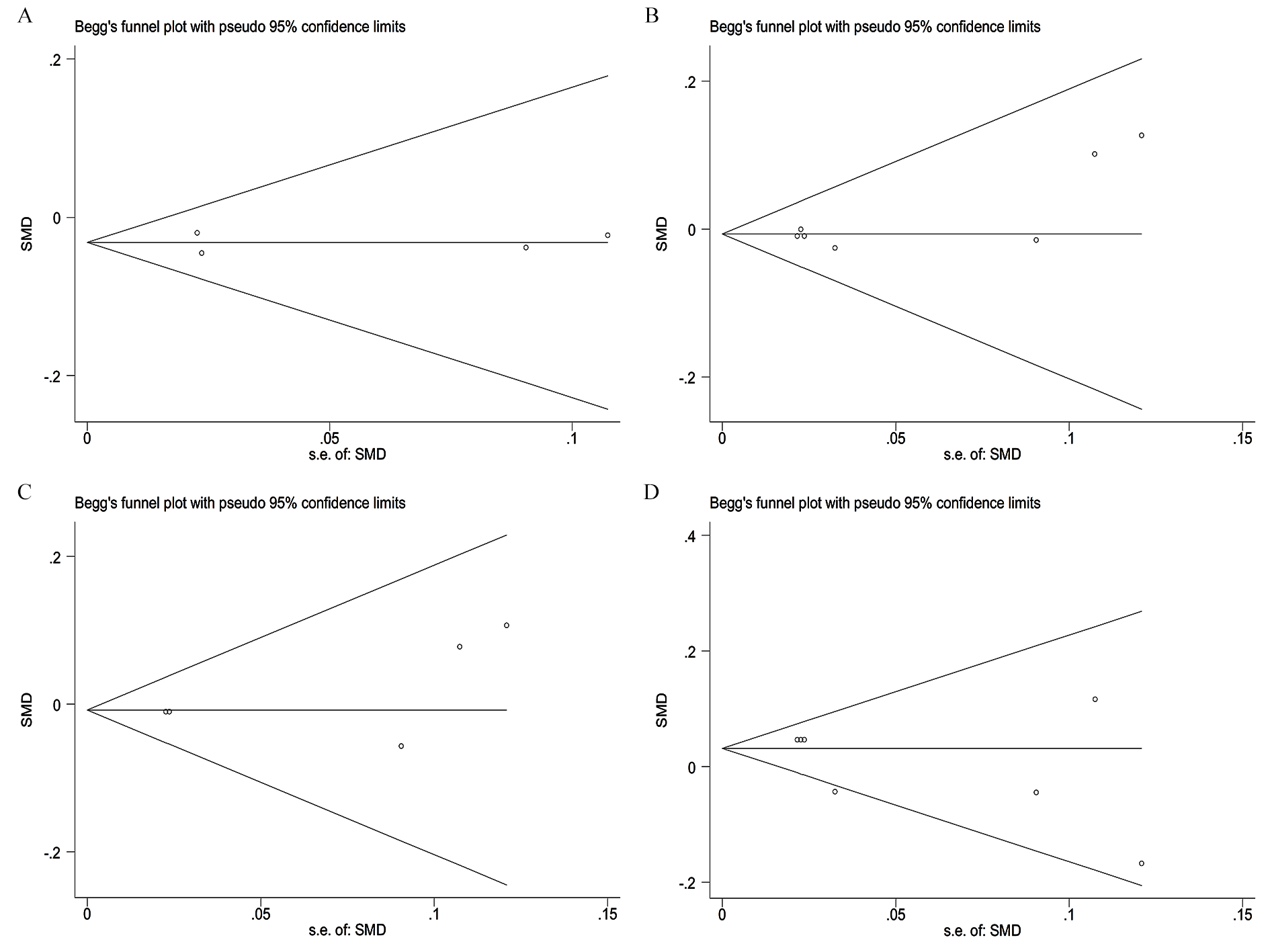


**Figure S11.** Begg’s funnel plot of the effects of lncRNA rs10757274 variant and circulating lipid levels. Each small circle represents a separate study, the diverging lines represent 95% CI and the central line is SMD. [A: rs10757274 on TG levels (*P* = 0.20); B: rs10757274 on TC levels (*P* = 0.08); C: rs10757274 on LDL-C levels (*P* = 0.26); D: rs10757274 on HDL-C levels (*P* = 0.09)]


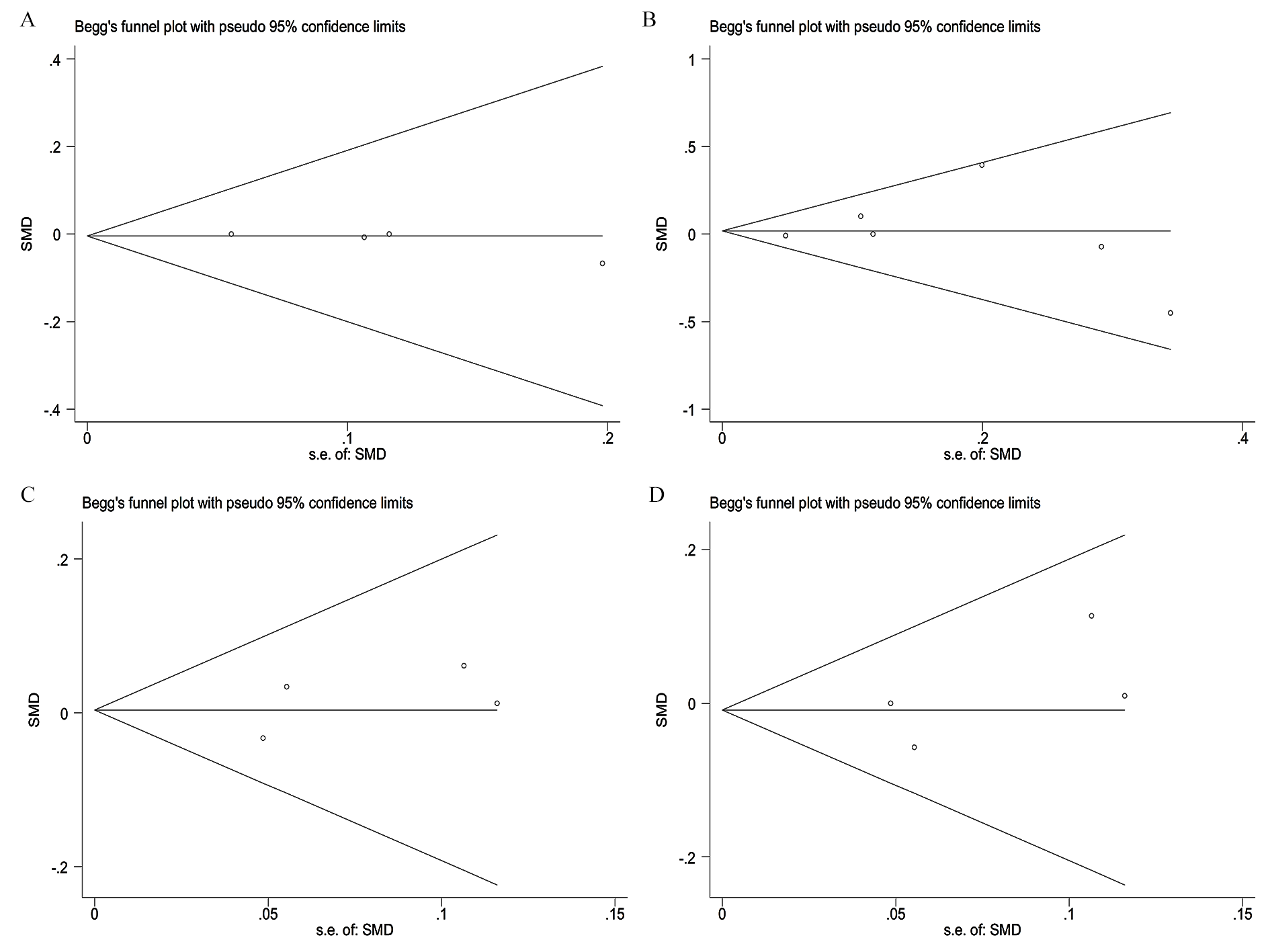


**Figure S12.** Begg’s funnel plot of the effects of lncRNA rs10757278 variant and circulating lipid levels. Each small circle represents a separate study, the diverging lines represent 95% CI and the central line is SMD. [A: rs10757278 on TG levels (*P* = 0.09); B: rs10757278 on TC levels (*P* = 0.98); C: rs10757278 on LDL-C levels (*P* = 0.52); D: rs10757278 on HDL-C levels (*P* = 0.39)]


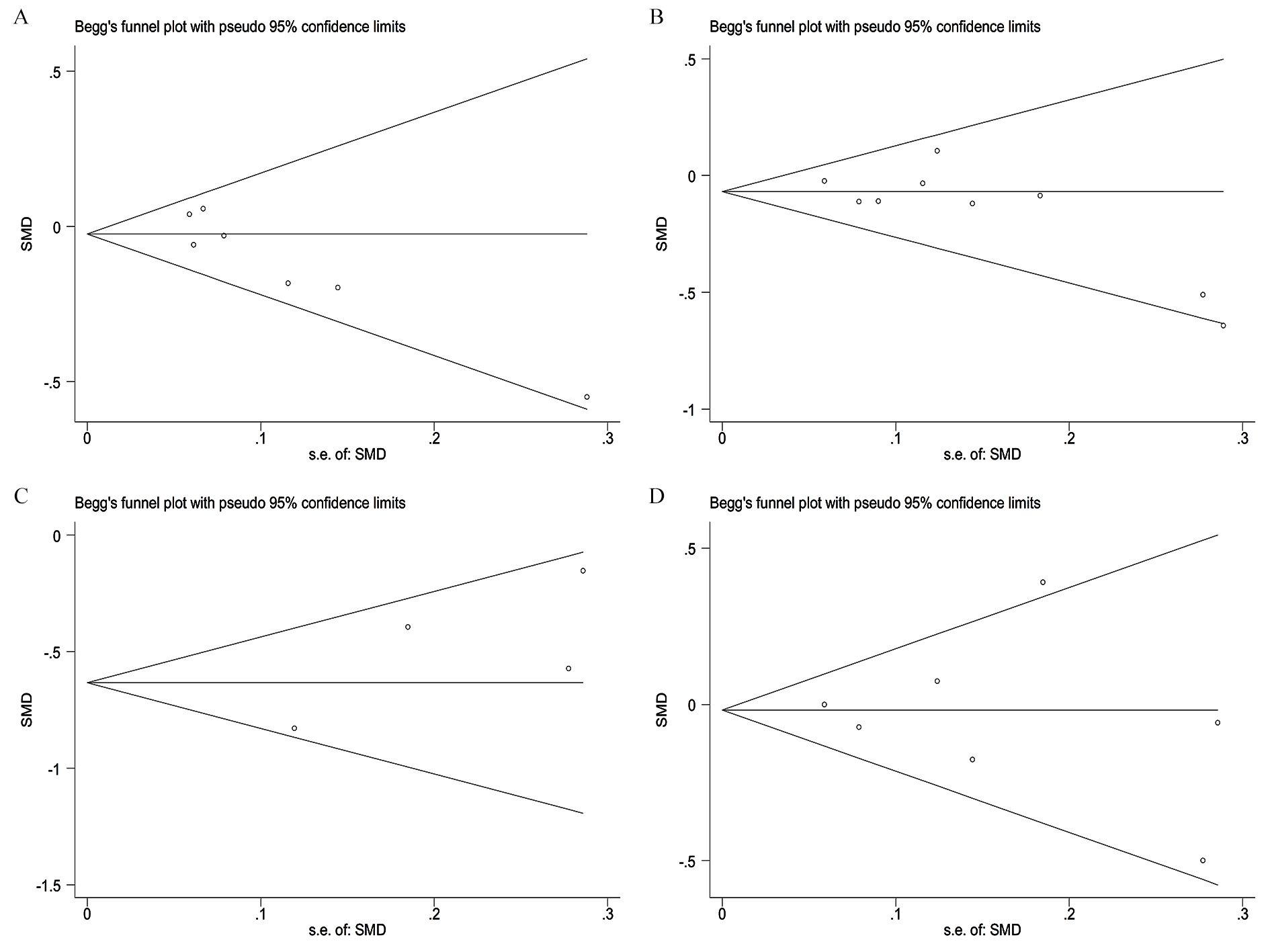


**Figure S13.** Begg’s funnel plot of the effects of CDKN2A/2B rs10811661 variant and circulating lipid levels. Each small circle represents a separate study, the diverging lines represent 95% CI and the central line is SMD. [A: rs10811661 on TG levels (*P* = 0.07); B: rs10811661 on TC levels (*P* = 0.40); C: rs10811661 on LDL-C levels (*P* = 0.17); D: rs10811661 on HDL-C levels (*P* = 0.97)]

**References of included studies**

R1. Yayla Ç, Okyay K, Yılmaz A, Şahinarslan A, Yar Sağlam AS, Eyiol A, Bolayır HA, Sezenöz B, Menevşe S, Çengel A. Association of rs10757274 and rs2383206 Polymorphisms on 9p21 locus with Coronary Artery Disease in Turkish Population. Korean Circ J. 2016;46:615-621.

R2. Ye S, Willeit J, Kronenberg F, Xu Q, Kiechl S. Association of genetic variation on chromosome 9p21 with susceptibility and progression of atherosclerosis: a population-based, prospective study. J Am Coll Cardiol. 2008;52:378-84.

R3. Brautbar A, Ballantyne CM, Lawson K, Nambi V, Chambless L, Folsom AR, Willerson JT, Boerwinkle E. Impact of adding a single allele in the 9p21 locus to traditional risk factors on reclassification of coronary heart disease risk and implications for lipid-modifying therapy in the Atherosclerosis Risk in Communities study. Circ Cardiovasc Genet. 2009;2:279-85.

R4. Yamagishi K, Folsom AR, Rosamond WD, Boerwinkle E; ARIC Investigators. A genetic variant on chromosome 9p21 and incident heart failure in the ARIC study. Eur Heart J. 2009;30:1222-8.

R5. Peng WH, Lu L, Zhang Q, Zhang RY, Wang LJ, Yan XX, Chen QJ, Shen WF. Chromosome 9p21 polymorphism is associated with myocardial infarction but not with clinical outcome in Han Chinese. Clin Chem Lab Med. 2009;47:917-22.

R6. Wen J, Rönn T, Olsson A, Yang Z, Lu B, Du Y, Groop L, Ling C, Hu R. Investigation of type 2 diabetes risk alleles support CDKN2A/B, CDKAL1, and TCF7L2 as susceptibility genes in a Han Chinese cohort. PLoS One. 2010;5:e9153.

R7. Patel RS, Su S, Neeland IJ, Ahuja A, Veledar E, Zhao J, Helgadottir A, Holm H, Gulcher JR, Stefansson K, Waddy S, Vaccarino V, Zafari AM, Quyyumi AA. The chromosome 9p21 risk locus is associated with angiographic severity and progression of coronary artery disease. Eur Heart J. 2010;31:3017-23.

R8. Ye S, Willeit J, Xiao Q, Motterle A, Laxton RC, Oberhollenzer F, Kiechl S, Xu Q. Single nucleotide polymorphism on chromosome 9p21 and endothelial progenitor cells in a general population cohort. Atherosclerosis. 2010;208:451-5.

R9. Dandona S, Stewart AF, Chen L, Williams K, So D, O'Brien E, Glover C, Lemay M, Assogba O, Vo L, Wang YQ, Labinaz M, Wells GA, McPherson R, Roberts R. Gene dosage of the common variant 9p21 predicts severity of coronary artery disease. J Am Coll Cardiol. 2010;56:479-86.

R10. Nambi V, Boerwinkle E, Lawson K, Brautbar A, Chambless L, Franeschini N, North KE, Virani SS, Folsom AR, Ballantyne CM. The 9p21 genetic variant is additive to carotid intima media thickness and plaque in improving coronary heart disease risk prediction in white participants of the Atherosclerosis Risk in Communities (ARIC) Study. Atherosclerosis. 2012;222:135-7.

R11. Plichart M, Empana JP, Lambert JC, Amouyel P, Tiret L, Letenneur L, Berr C, Tzourio C, Ducimetière P. Single polymorphism nucleotide rs1333049 on chromosome 9p21 is associated with carotid plaques but not with common carotid intima-media thickness in older adults. A combined analysis of the Three-City and the EVA studies. Atherosclerosis. 2012;222:187-90.

R12. Gioli-Pereira L, Santos PC, Ferreira NE, Hueb WA, Krieger JE, Pereira AC. Higher incidence of death in multi-vessel coronary artery disease patients associated with polymorphisms in chromosome 9p21. BMC Cardiovasc Disord. 2012;12:61.

R13. Erridge C, Gracey J, Braund PS, Samani NJ. The 9p21 locus does not affect risk of coronary artery disease through induction of type 1 interferons. J Am Coll Cardiol. 2013;62:1376-81.

R14. Gong L, Chen J, Lu J, Fan L, Huang J, Zhang Y, Lv B, Hui R, Wang Y. The 9p21 locus is associated with coronary artery disease and cardiovascular events in the presence (but not in the absence) of coronary calcification. PLoS One. 2014;9:e94823.

R15. Hindy G, Ericson U, Hamrefors V, Drake I, Wirfält E, Melander O, Orho-Melander M. The chromosome 9p21 variant interacts with vegetable and wine intake to influence the risk of cardiovascular disease: a population based cohort study. BMC Med Genet. 2014;15:1220.

R16. Lee IT, Goodarzi MO, Lee WJ, Rotter JI, Chen YD, Liang KW, Lee WL, Sheu WH. The chromosome 9p21 variant not predicting long-term cardiovascular mortality in Chinese with established coronary artery disease: an eleven-year follow-up study. Biomed Res Int. 2014;2014:626907.

R17. Lara-Riegos JC, Ortiz-López MG, Peña-Espinoza BI, Montúfar-Robles I, Peña-Rico MA, Sánchez-Pozos K, Granados-Silvestre MA, Menjivar M. Diabetes susceptibility in Mayas: Evidence for the involvement of polymorphisms in HHEX, HNF4alpha, KCNJ11, PPARgamma, CDKN2A/2B, SLC30A8, CDC123/CAMK1D, TCF7L2, ABCA1 and SLC16A11 genes. Gene. 2015;565:68-75.

R18. Chen Y, Zhao Y, Liu GF. Associations of polymorphisms of SLC30A8, CDKN2A/2B, HHEX and TCF7L2 gene with type 2 diabetes mellitus in Chinese Han population in northeast China. Chinese Journal of Gerontology. 2015,35:4201-4204.

R19. Bi J, Yang L, Liu D, Wu J, Tong X, Cen S, Zhou D, Zhang T, Yi L. Sequence variants on chromosome 9p21 are associated with ischemic stroke and the lipids level in Chinese Han population. J Stroke Cerebrovasc Dis. 2015;24:894-900.

R20. Abid K, Mili D, Kenani A. Polymorphism on Chromosome 9p21.3 Is Associated with Severity and Early-Onset CAD in Type 2 Diabetic Tunisian Population. Dis Markers. 2015;2015:792679.

R21. Lv J, Tang OS. The association between rs4977574 poIymorphism at chromosome 9p21 and coronary artery disease. Journal of Electrocardiology and Circulation. 2015,34:437-439.

R22. Matsuoka R, Abe S, Tokoro F, Arai M, Noda T, Watanabe S, Horibe H, Fujimaki T, Oguri M, Kato K, Minatoguchi S, Yamada Y. Association of six genetic variants with myocardial infarction. Int J Mol Med. 2015;35:1451-9.

R23. Lee IT, Liang KW, Wang JS, Lee WJ, Chen YI, Lin SY, Lee WL, Sheu WH. Value of Chromosome 9p21 Polymorphism for Prediction of Cardiovascular Mortality in Han Chinese Without Coronary Lesions: An Observational Study. Medicine (Baltimore). 2015;94:e1538.

R24. Beigi SS, Ghaderian SM, Doosti A. Investigation of the Association between rs4977574 A > G Polymorphism in ANRIL Gene and Coronary Artery Disease in Iranian Population. Int Cardiovasc Res J. 2015;9:139-144.

R25. Phani NM, Adhikari P, Nagri SK, D'Souza SC, Satyamoorthy K, Rai PS. Replication and Relevance of Multiple Susceptibility Loci Discovered from Genome Wide Association Studies for Type 2 Diabetes in an Indian Population. PLoS One. 2016;11:e0157364.

R26. Zhao Q, Liao S, Wei H, Liu D, Li J, Zhang X, Yan M, Jin T. CDKN2BAS polymorphisms are associated with coronary heart disease risk a Han Chinese population. Oncotarget. 2016;7:82046-82054.

R27. Zheng C, Yang H, Wang Q, Rao H, Diao Y. Association analysis of five SNP variants with gout in the Minnan population in China. Turk J Med Sci. 2016;46:361-7.

R28. Lee CJ, Lee JY, Oum CY, Youn JC, Kang SM, Choi D, Jang Y, Park S, Jee SH, Lee SH. The Effect of FLT1 Variant on Long-Term Cardiovascular Outcomes: Validation of a Locus Identified in a Previous Genome-Wide Association Study. PLoS One. 2016;11:e0164705.

R29. Shendy HA, Hassanein SI, Gad MZ. "Desert" gene (Chr9p21) variants as novel markers for coronary artery disease. Anatol J Cardiol. 2017;18:84-89.

R30. Tang O, Lv J, Cheng Y, Qin F. The Correlation Between 9p21 Chromosome rs4977574 Polymorphism Genotypes and the Development of Coronary Artery Heart Disease. Cardiovasc Toxicol. 2017;17:185-189.

R31. Liu J, Wang SZ, Wang QL, Du JG, Wang BB. The Correlation Between Blood lipid,Blood Glucose Levels of Gestational Diabetes Mellitus and CDKN2A/2B Gene Polymorphism. Labeled Immunoassays&Clin Med. 2018, 25: 1360-1363.

R32. Mehramiz M, Ghasemi F, Esmaily H, Tayefi M, Hassanian SM, Sadeghzade M, Sadabadi F, Moohebati M, Azarpazhooh MR, Parizadeh SMR, Heidari-Bakavoli A, Safarian M, Nematy M, Ebrahimi M, Ryzhikov M, Ferns GA, Ghayour-Mobarhan M, Avan A. Interaction between a variant of CDKN2A/B-gene with lifestyle factors in determining dyslipidemia and estimated cardiovascular risk: A step toward personalized nutrition. Clin Nutr. 2018;37:254-261.

R33. Plengvidhya N, Chanprasert C, Chongjaroen N, Yenchitsomanus PT, Homsanit M, Tangjittipokin W. Impact of KCNQ1, CDKN2A/2B, CDKAL1, HHEX, MTNR1B, SLC30A8, TCF7L2, and UBE2E2 on risk of developing type 2 diabetes in Thai population. BMC Med Genet. 2018;19:93.

R34. Mahdavi S, Jenkins DJA, El-Sohemy A. Genetic variation in 9p21 is associated with fasting insulin in women but not men. PLoS One. 2018;13:e0202365.

R35. Shahid SU, Shabana NA, Rehman A, Humphries S. GWAS implicated risk variants in different genes contribute additively to increase the risk of coronary artery disease (CAD) in the Pakistani subjects. Lipids Health Dis. 2018;17:89.

R36. Kunnas T, Piesanen J, Nikkari ST. Association of a Chromosome Locus 9p21.3 CDKN2B-AS1 Variant rs4977574 with Hypertension: The TAMRISK Study. Genet Test Mol Biomarkers. 2018;22:327-330.

R37. Temel ŞG, Ergören MÇ. The association between the chromosome 9p21 CDKN2B-AS1 gene variants and the lipid metabolism: A pre-diagnostic biomarker for coronary artery disease. Anatol J Cardiol. 2019;21:31-38.

R38. Shakhtshneider E, Orlov P, Semaev S, Ivanoshchuk D, Malyutina S, Gafarov V, Ragino Y, Voevoda M. Analysis of Polymorphism rs1333049 (Located at 9P21.3) in the White Population of Western Siberia and Associations with Clinical and Biochemical Markers. Biomolecules. 2019;9:290.

R39. Leu HB, Chung CM, Chen JW, Pan WH. The Mediterranean diet reduces the genetic risk of chromosome 9p21 for myocardial infarction in an Asian population community cohort. Sci Rep. 2019;9:18405.

R40. Shakhtshneider E, Orlov P, Semaev S, Ivanoshchuk D, Maksimov V, Ragino Y, Malyutina S, Voevoda M. RS1333049 AND LIPID PROFILE IN RUSSIA POPULATION. Atherosclerosis. 2019, 287: e192.

R41. Jacobson P, Peltonen M, Svensson PA, Taube M, Andersson-Assarsson JC, Sjoholm K, Bouchard C, Carlsson B, Carlsson LMS. 9p21.3 Coronary Artery Disease Locus Identifies Patients With Treatment Benefit From Bariatric Surgery in the Nonrandomized Prospective Controlled Swedish Obese Subjects Study. Circ Genom Precis Med. 2020;13:460-465.

R42. He SQ, Yang M, Yang Y, Wang FY, Wang XL, Lu TL, Jue M, Li YP. Correlation of single nucleotide polymorphisms in miR-129 and CDKN2B-AS1 with type 2 diabetes risk in a Chinese Han population in Yunnan province. JOURNAL OF GUIZHOU MEDICAL UNIVERSITY. 2021; 46: 497-510.

R43. Bogari N, Dannoun A, Athar M, Elkhateeb O, Porqueddu M, Allam R, Alamanni F. Genetic Association of rs10757278 on Chromosome 9p21 and Coronary Artery Disease in a Saudi Population. Int J Gen Med. 2021;14:1699-1707.
